# Quantile forecast combination using stochastic dominance

**DOI:** 10.1007/s00181-017-1343-1

**Published:** 2017-10-14

**Authors:** Mehmet Pinar, Thanasis Stengos, M. Ege Yazgan

**Affiliations:** 1 Business School, Edge Hill University, St Helens Road, Ormskirk, Lancashire L39 4QP UK; 2 Department of Economics, University of Guelph, Guelph, N1G 2W1 Canada; 3 Department of Economics, Istanbul Bilgi University, 34060 Istanbul, Turkey

**Keywords:** Nonparametric stochastic dominance, Mixed integer programming, Forecast combinations, C12, C13, C14, C15, G01

## Abstract

This paper derives optimal forecast combinations based on stochastic dominance efficiency (SDE) analysis with differential forecast weights for different quantiles of forecast error distribution. For the optimal forecast combination, SDE will minimize the cumulative density functions of the levels of loss at different quantiles of the forecast error distribution by combining different time-series model-based forecasts. Using two exchange rate series on weekly data for the Japanese yen/US dollar and US dollar/Great Britain pound, we find that the optimal forecast combinations with SDE weights perform better than different forecast selection and combination methods for the majority of the cases at different quantiles of the error distribution. However, there are also some very few cases where some other forecast selection and combination model performs equally well at some quantiles of the forecast error distribution. Different forecasting period and quadratic loss function are used to obtain optimal forecast combinations, and results are robust to these choices. The out-of-sample performance of the SDE forecast combinations is also better than that of the other forecast selection and combination models we considered.

## Introduction

Since the seminal work of Bates and Granger (1969), combining the forecasts of different models, rather than relying on the forecasts of individual models, has come to be viewed as an effective way to improve the accuracy of predictions regarding a certain target variable. A significant number of theoretical and empirical studies, e.g., Timmermann (2006) and Stock and Watson (2004), have been able to demonstrate the superiority of combined forecasts over single-model-based predictions.

In this context, the central question is to determine the optimal weights used in the calculation of combined forecasts. In combined forecasts, the weights attributed to each model depend on the model’s out-of-sample performance. Over time, the forecast errors used for the calculation of optimal weights change; thus, the weights themselves vary over time. However, in empirical applications, numerous papers (Clemen 1989; Stock and Watson 1999a, b, 2004; Hendry and Clements 2004; Smith and Wallis 2009; Huang and Lee 2010; Aiolfi et al. 2011; Geweke and Amisano 2012) have found that equally weighted forecast combinations often outperform or perform almost as well as estimated optimal forecast combinations. This finding is frequently referred as the “forecast combination puzzle” by Stock and Watson (2004) because the efficiency cost of estimating the additional parameters of an optimal combination exceeds the variance reduction gained by deviating from equal weights.^1^ Overall, even though different optimal forecast combination weights are derived for static, dynamic, or time-varying situations, most empirical findings suggest that the simple average forecast combination outperforms forecast combinations with more sophisticated weighting schemes.

In this paper, we will follow an approach for the combination of forecasts based on stochastic dominance (SD) analysis, and we test whether a simple average combination of forecasts would outperform forecast combinations with more elaborate weights. In this context, we will examine whether an equally weighted forecast combination is optimal when we analyze the forecast error distribution. Rather than assigning arbitrary equal weights to each forecast, we use stochastic dominance efficiency (SDE) analysis to propose a weighting scheme that dominates the equally weighted forecast combination.

Typically, SD comparisons are conducted in a pair-wise manner. Barrett and Donald (2003) developed pair-wise SD comparisons that relied on Kolmogorov–Smirnov-type tests developed within a consistent testing environment. This offers a generalization of Beach and Davidson (1983), Anderson (1996), and Davidson and Duclos (2000), who examined second-order SD using tests that rely on pair-wise comparisons made at a fixed number of arbitrarily chosen points, an undesirable feature that may lead to a test inconsistency. Linton et al. (2005) propose a subsampling method that can address both dependent samples and dependent observations within samples. This is appropriate for conducting SD analysis for model selection among many forecasts. In this context, comparisons are available for pairs for which one can compare one forecast with another forecast and conclude whether one forecast dominates the other. Hence, one can find the best individual model by comparing all forecasts. In this case, the dominant model (optimal one) will always produce a distribution of forecast errors that is lower than the distribution of forecast errors obtained from another forecast model. Pair-wise dominance would suggest that the optimal model will always produce a lower number of errors above all given error levels than any other model. Lately, multivariate (multidimensional) comparisons have become more popular. Multivariate SD comparisons in the finance literature led to the development of SD efficiency testing methodologies first discussed by Fishburn (1977). In line with Fishburn (1977), Post (2003) provided a SD efficiency testing approach to test market efficiency by allowing full weight diversification across different assets. Recently, Scaillet and Topaloglou (2010), ST hereafter, used SD efficiency tests that can compare a given portfolio with an optimally diversified portfolio constructed from a set of assets.^2^ The recent testing literature in finance examines whether a given weighted combination of assets dominates the market at all return levels. In this paper, we adapt the SDE methodology into a forecasting setting to obtain the optimal forecast combination. The main contribution of the paper is the derivation of an optimal forecast combination based on SDE analysis with differential forecast weights. For the optimal forecast combination, this forecast combination will minimize the number of forecast errors that surpass a given threshold level of loss. In other words, we will examine the forecast error distribution of the average forecast combination at different parts of the empirical distribution and test whether the average forecast combination is optimal at different sections of the forecast error distribution. Furthermore, we investigate whether there is an alternative forecast combination that can offer an optimal forecast combination at some parts of the forecast error distribution.

The mainstream forecast combination literature obtains the forecast combination weights through the minimization of the total sum of the squared forecast errors (or the mean squared forecast errors) taking into account all the forecasts over the whole period. For instance, the seminal paper of Granger and Ramanathan (1984) employs ordinary least squares (minimizing the sum of squared errors) to obtain optimal weights for the point forecasts of individual models. The forecast combination literature also consists of methods that analyze the optimal forecast combinations based on quantiles of the forecasts (see e.g., Taylor and Bunn 1998; Giacomini and Komunjer 2005; Clements et al. 2008; Gerlach et al. 2011). In that context for example, Giacomini and Komunjer (2005) obtain forecast weights based on a generalized methods of moments (GMM) estimation approach conditional on quantile forecasts. In a standard quantile regression setting, when the quadratic loss function is replaced with the absolute loss function, individual point forecasts are used to minimize the absolute forecast errors for a given quantile level (Koenker 2005). In that case, if the absolute forecast errors are considered from the whole distribution, this leads to a quantile regression for the median (see, e.g., Nowotarski et al. 2014). Our approach differs from the above-mentioned mainstream forecast combinations, and it is complementary to them. In particular, methods that minimize the sum of the squared forecast errors find forecast combinations that work well at the center of the distribution. However, different forecast combinations might work better at different areas of the empirical distribution of the forecast errors if the loss function or forecast error distribution is skewed (see, e.g., Elliott and Timmermann 2004). Similarly, quantile regressions minimize the absolute forecast errors (or mean absolute forecast errors) based on given quantile forecasts. This objective function (similar to that of sum of squared forecast errors) is set to minimize a single measure, such as the mean absolute forecast errors up to a given quantile; however, it ignores how the absolute forecast errors are distributed up to the given quantile. In this context, our paper analyzes the entire forecast error distribution, which takes into account all moments. Rather than relying on single optimal forecast combinations, we derive the optimal forecast combinations at different parts of the empirical forecast error distribution. In other words, rather than choosing the one forecast combination that minimizes the mean squared forecast errors (or mean absolute forecast errors), we derive different combinations that will maximize the cumulative distribution function (cdf) of forecast errors up to a given threshold level. In this respect, SDE method does not provide the lowest mean absolute forecast error at a given quantile; however, it provides the lowest number of forecast errors above a given threshold level.

In order to better understand the distinction between the two approaches, one relying on minimizing the number of forecast errors above a given threshold and the other minimizing the overall squared forecast errors (or absolute forecast errors) for a given quantile, we provide a brief discussion on how SDE methodology complements the mainstream forecast combinations. Forecasters and investors follow a certain strategy and depending on their risk attitudes they try to minimize their losses or forecast errors. Some might consider to minimize the forecast errors for all possible forecast levels, and as such, they minimize the total sum of (squared) forecast errors (e.g., MSFE). Others might want to try to minimize the forecast errors for a given quantile of forecasts (quantile regression). On the other hand, there may be a forecaster (like an insurance company) who compensates above a given threshold level of loss. In that case, the company in question would offer a guarantee to compensate their customers if their forecast error (loss) is above a given level. Hence, this company would like to minimize the forecast errors (losses) that are above this threshold so that to minimize its compensation levels, something that may not be achieved by minimizing the total sum of squared forecast errors (or the absolute forecast errors for this quantile). The latter methods will minimize the overall loss (or quantile loss), but the number of losses above a given threshold level might not be the lowest as derived by the SDE approach. In that context, the SDE methodology is designed to combine forecasts that minimizes the number of forecast errors above a given threshold, and this is obtained by maximizing the empirical cumulative distance between the loss generated by the equally weighted forecasts and the alternative one for this threshold loss level. Therefore, the SDE method produces a forecast combination that complements the more conventional forecast selection and combination methods and can serve forecasters and investors to obtain better forecast combinations depending on their strategy and policy.

We use two exchange rate series given in a weekly frequency for the Japanese yen/US dollar and US dollar/Great Britain pound to derive optimal forecast combinations with the SDE methodology for different forecasting periods (during and after the 2007/2009 financial crisis) and for different forecast horizons. Overall, we find that the optimal forecast combinations with SDE weights perform better than different forecast selection and combination methods for the majority of the cases. However, there are also some very few cases where some other forecast selection and combination model performs equally well at some parts of the forecast error distribution. For the optimal forecast combination obtained with SDE weights, the best forecasting model (i.e., the model that gets relatively more weight than other forecasting models) includes different sets of models at different parts of the empirical distribution. On average, autoregressive and self-exciting threshold autoregressive models are the main contributors to the optimal forecast combination for both the Japanese yen/US dollar and US dollar/Great Britain pound exchange rate application, and during and after the 2007/2009 financial crisis.

The remainder of the paper includes the following. In Sect. 2, we define the concept of SDE and discuss the general hypothesis for SDE at any order. Section 3 describes the data, time-series forecasting models and forecast methods used in our paper as well as alternative forecast selection and combination methods. Section 4 presents the empirical analysis where we use the SDE methodology to find the optimal forecast combination for the two exchange rate series for different forecast periods with different forecast horizons and compare these findings with those from the other forecast selection and combination methods. Section 5 offers robustness analysis, and finally, Sect. 6 concludes.

## Hypothesis, test statistics and asymptotic properties

Let us start with data $\left\{ y_{t};t\in \mathbb {Z}\right\}$ and the $\left( m\times 1\right)$ column vector of forecasts $\left\{ \widehat{\varvec{y}}_{t+h,t};t,h\in \mathbb {Z}\right\}$ for $y_{t+h}$ obtained from *m* different forecasting models generated at time *t* for the period of $t+h$ ($h\ge 1$), where *h* is the forecast horizon and *T* is the final forecasting period. Furthermore, let $y_{t+h}$ denote the actual values over the same forecast period.

The equally weighted column vector, $\varvec{\tau }$, is used to obtain the simple average of individual forecasts derived from the *m* different models, i.e., $\widehat{y}_{t+h,t}^{ew}=\varvec{\tau }^{^{\prime }} \widehat{\varvec{y}}_{t+h,t}$, where $\varvec{\tau }$ is the $\left( m\times 1\right)$ column vector with entries $\frac{1}{m}$’s. Forecast errors with the equally weighted forecast combination are obtained by $\varepsilon _{t+h,t}^{ew}=y_{t+h}-\widehat{y}_{t+h,t}^{ew}$. Let us now consider an alternative weighting column vector $\varvec{\lambda }\in \mathbb {L}$, where $\mathbb {L}:=\{\varvec{\lambda }\in \mathbb {R}_{+}^{n}:\varvec{e}^{\prime }\varvec{\lambda }=1\}$ with *e* being a vector of ones. With this alternative weighting scheme, one can obtain a forecast combination, i.e., $\widehat{y}_{t+h,t}^{w}=\varvec{\ \lambda }^{\prime }\widehat{\varvec{y}}_{t+h,t}$. Similarly, forecast errors with this alternative weighting scheme are obtained by $\varepsilon _{t+h,t}^{w}=y_{t+h}-\widehat{y}_{t+h,t}^{w}$.

For this paper, we follow a loss function that depends on the forecast error, i.e., $L(\varepsilon _{t+h,t})$, that has the following properties (Granger 1999):

*i*.: $$L(0)=0,$$
*ii*.: $$\underset{e}{\min }L(\varepsilon )=0,$$ i.e., $$L(\varepsilon )\ge 0,$$
*iii*.: $L(\varepsilon )$ is monotonic non-decreasing as $\varepsilon$ moves away from 0:

i.e., $L(\varepsilon _{1})\ge L(\varepsilon _{2})$ if $\varepsilon _{1}>\varepsilon _{2}\ge 0$ and if $\varepsilon _{1}<\varepsilon _{2}\le 0$.

(*i*) suggests that there is no loss when there is no error, (*ii*) suggests that the minimum loss is zero, and finally, (*iii*) suggests that the loss is determined by its distance to zero error irrespective of its sign.^3^ This loss function may have further assumptions, such as being symmetric, homogenous, or differentiable up to some order (see Granger 1999, for the details). Hence, the associated loss functions with the equally weighted forecast combination and forecast combination with alternative weighting scheme are $L(\varepsilon _{t+h,t}^{ew})$ (i.e., $L(y_{t+h}-\varvec{\tau }^{\prime }\widehat{\varvec{y}}_{t+h,t})$) and $L(\varepsilon _{t+h,t}^{w})$ (i.e., $L(y_{t+h}-\varvec{\lambda }^{\prime }\widehat{\varvec{y}}_{t+h,t})$), respectively.

Note that we can have different forecast errors depending on the different choices of weights available to combine forecasts. The forecast combination literature employs various objective functions derived from the loss function to obtain optimal weights to combine forecasts (see, e.g., Hyndman and Koehlerb 2006, for an extensive list of accuracy measures). It is common in the literature to use the norm of the loss function based on forecast errors to find the optimal weights (see Timmermann 2006).

In other words, the most common way of obtaining the optimal vector of combination weights, $\varvec{\lambda }_{t+h,t}^{*}$, is given by solving the problem

1 $$\begin{aligned} \varvec{\lambda }_{t+h,t}^{*}=\underset{\varvec{\lambda }}{\arg \min }E\left[ L(\varepsilon _{t+h,t}(\varvec{\lambda }_{t+h,t}))\mid \widehat{\varvec{y}}_{t+h,t}\right] \qquad s.t.\qquad \varvec{e} ^{\prime }\varvec{\lambda }=1, \end{aligned}$$

where the expectation is taken over the conditional distribution of $\varepsilon _{t+h,t}$. Similarly, the loss function might be based on quadratic loss function (see, e.g., Elliott and Timmermann 2004).

However, it is well known that all of the moments of the forecast error distribution will affect the combination of weights (see, e.g., Geweke and Amisano 2011), and if one were to find the optimal weights by analyzing the entire distribution of the errors, this would lead to a more informative outcome. In this paper, SDE analysis allows for all moments to be considered as it examines the entire forecast error distribution. For example, if one were to find weights by minimizing the mean squared forecast errors (MSFE) and the forecast distribution was asymmetric with some important outliers, then the weighted forecast combination, which would have been obtained as the solution, would have ignored these important features of the empirical distribution. In other words, under an MSFE loss function (i.e., quadratic loss function), the optimal forecast combination is obtained by the optimal trade-off between squared bias and the forecast error variance (i.e., the optimal forecast combination only depends on the first two moments of the forecast errors). However, if the forecast error distribution is skewed, different weighted forecast combinations would work better at different parts of the empirical distribution of the forecast errors (see, e.g., Elliott and Timmermann 2004). Hence, looking at all of the moments of the forecast error would result in more robust weighting schemes. In the case of asymmetric loss and nonlinearities, optimal weights based on the general loss functions that rely on first and second moment of the forecast errors are not robust (see e.g., Patton and Timmermann 2007). In this paper, rather than the loss function that relies on only two moments, we analyze the full empirical distribution of the loss which incorporates information beyond the first two moments. One could obtain optimal forecast combination for different sections of the distribution rather than single forecast combination where the latter case might work well in some sections of the loss distribution and worse in other parts, whereas, in our case, one could obtain various combinations which would work well for at different sections of the error distribution and one could choose which combination to use. Our approach is also a nonparametric one that does not rely on assumptions as its criteria do not impose explicit functional form requirements on individual preferences or restrictions on the functional forms of probability distributions since we are analyzing the full distribution of the loss (i.e., magnitude of the forecast error distribution).

In short, the quadratic loss function minimizes the sum of squared forecast errors (or mean squared forecast errors) and the quantile regression minimizes the sum of absolute errors (or mean absolute errors) for a given quantile. If one were to minimize the squared forecast errors by looking at the whole distribution (or quantile), these approaches could be appropriate. On the other hand, with the SDE methodology one minimizes the number of forecast errors (or squared forecast errors) above a given threshold error level. In that respect, SDE approach complements the existing forecast selection and/or combination methods when one’s priority is to minimize the number of forecasts above a given threshold. For example, this could be the case, when a company promises to compensate its consumers if their forecasts give errors that are above a threshold error level. Standard approaches would minimize an overall single measure (mean squared forecast error or mean absolute error for a given quantile). However, these objective functions are not designed to minimize the number of errors above a given threshold error level and might produce a higher number of losses above this given threshold. In this respect, SDE offers a complementary approach to forecast combination if the number of losses above a threshold is deemed more important than the overall (or quantile) loss.

In this paper, we test whether the cumulative distribution function (cdf) of the loss function with the equally weighted forecast combination is stochastically efficient or not. $F(L(\varepsilon _{t+h,t}^{ew}))$ and $F(\,L(\varepsilon _{t+h,t}^{w}))$ are the continuous cdf of the $L(\varepsilon _{t+h,t}^{ew})$ and $L(\varepsilon _{t+h,t}^{w})$ with weights $\varvec{\tau }$ (equal weights) and $\varvec{\lambda }$ (alternative weights). Furthermore, $G(z,\varvec{\tau };F)$ and $G(z, \varvec{\lambda };F)$ the cdf’s of the loss functions associated with the forecast combinations of $\varvec{\tau }^{^{\prime }}\widehat{ \varvec{y}}_{t+h,t}$ and $\varvec{\lambda }^{^{\prime }}\widehat{ \varvec{y}}_{t+h,t}$ at point *z* given $\displaystyle G(z,\varvec{\ \tau };F):=\int _{\mathbb {R}^{n}}\mathbb {I}\{L(\varepsilon _{t+h,t}^{ew})\le z\}dF(L(\varepsilon _{t+h,t}))$ and $\displaystyle G(z,\varvec{\lambda } ;F):=\int _{\mathbb {R}^{n}}\mathbb {I}\{L(\varepsilon _{t+h,t}^{w})\le z\}dF(L(\varepsilon _{t+h,t}))$, respectively, where *z* represents the level of loss^4^ and $\mathbb {I}$ represents the indicator function (Davidson and Duclos 2000).

For any two forecast combinations, we say that the forecast combination $\varvec{\lambda }^{^{\prime }}\widehat{\varvec{y}}_{t+h,t}$ dominates the distribution of the equally weighted forecast combination $\varvec{\tau }^{^{\prime }}\widehat{\varvec{y}}_{t+h,t}$ stochastically at first order (SD1) if, for any point *z* of the loss distribution, $\displaystyle G(z,\varvec{\lambda };F)\ge \displaystyle G(z,\varvec{\tau };F)$.^5^ In the context of our analysis, if *z* denotes the loss level, then the inequality in the definition means that the proportion of loss obtained with the forecast combination of $\varvec{\lambda }^{^{\prime }}\widehat{\varvec{y}}_{t+h,t}$ at point *z* is no lower than the value (mass) of the cdf of the loss with the equally weighted forecast combination, $\varvec{\tau }^{^{\prime }} \widehat{\varvec{y}}_{t+h,t}$. In other words, the proportion of loss generated with the forecast combination of $\varvec{\lambda }^{^{\prime }}\widehat{\varvec{y}}_{t+h,t}$ above a given *z* level is less than the one with the equally weighted forecast combination, $\varvec{\tau } ^{^{\prime }}\widehat{\varvec{y}}_{t+h,t}$. If the forecast combination $\varvec{\lambda }^{^{\prime }}\widehat{\varvec{y}}_{t+h,t}$ dominates the equally weighted forecast combination $\varvec{\tau } ^{^{\prime }}\widehat{\varvec{y}}_{t+h,t}$ at the first order, then $\varvec{\lambda }^{^{\prime }}\widehat{\varvec{y}}_{t+h,t}$ yields the optimal forecast combination for that given loss level, *z*.

More precisely, to achieve stochastic dominance, we maximize the following objective function:

$$\begin{aligned} \underset{\varvec{\lambda }}{\text {Max}}\, \displaystyle [G(z, \varvec{\lambda };F)-\displaystyle G(z,\varvec{\tau };F)] \text {for a given}\, z \,\text {level}. \end{aligned}$$

This maximization results in the optimal forecast combination, $\varvec{ \ \lambda }^{^{\prime }}\widehat{\varvec{y}}_{t+h,t}$, that can be constructed from the set of forecast models in the sense that it reaches the minimum number of loss above a given loss level, *z*. In other words, $\varvec{\lambda }^{^{\prime }}\widehat{\varvec{y}}_{t+h,t}$ gives a combination that offers the highest number of forecast combinations that generates a loss that is below a given *z* level, and hence, it minimizes the number of forecasts that gives a loss above a given threshold, *z*.

The general hypotheses for testing whether the equally weighted forecast combination, $\varvec{\tau }^{^{\prime }}\widehat{\varvec{y}} _{t+h,t}$, is the optimal forecast combination at the stochastic dominance efficiency order of *j*, hereafter $SDE_{j}$, can be written compactly as:

$$\begin{aligned} H_{0}^{j}:&\mathcal {J}_{j}(z,\varvec{\lambda };F)\le \mathcal {J} _{j}(z, \varvec{\tau };F)\,\,\text{ for } \text{ given }\,z\in \mathbb {R}\, \text{ and }\, \text{ for } \text{ all }\, \varvec{\lambda }\in \mathbb {L}, \\ H_{1}^{j}:&\mathcal {J}_{j}(z,\varvec{\lambda };F)>\mathcal {J}_{j}(z, \varvec{\tau };F)\, \, \text{ for } \text{ given }\,z\in \mathbb {R}\, \text{ or }\, \text{ for } \text{ some }\, \varvec{\lambda }\in \mathbb {L}. \end{aligned}$$

where

2 $$\begin{aligned} \mathcal {J}_{j}(z,\varvec{\lambda };F)=\int _{\mathbb {R}^{n}}\frac{1}{ (j-1)!}(z-L(\varepsilon _{t+h,t}^{w}))^{j-1}\mathbb {I}\{L(\varepsilon _{t+h,t}^{w})\le z\}dF(L(\varepsilon _{t+h,t})) \end{aligned}$$

and $\mathcal {J}_{1}(z,\varvec{\lambda };F):=G(z,\varvec{\lambda } ;F)$. Under the null hypothesis $H_{0}^{j}$   there is no distribution of loss obtained from any alternative forecast combination $\varvec{\lambda }^{^{\prime }}\widehat{\varvec{y}}_{t+h,t}$ that dominates the loss distribution that is obtained from the equally weighted forecast combination at given level of loss, *z* level (i.e., a chosen quantile of loss level). In other words, under the null, we analyze whether the equally weighted forecast combination, $\tau ^{^{\prime }}\widehat{ \varvec{y}}_{t+h,t}$, is optimal at a given quantile of the loss distribution when compared to all possible combinations of forecasts **,**  $\varvec{\lambda }^{^{\prime }}\widehat{\varvec{y}}_{t+h,t}$, whereas under the alternative hypothesis $H_{1}^{j}$, we can construct a forecast combination $\varvec{\lambda }^{^{\prime }} \widehat{\varvec{y}}_{t+h,t}$ for which, for given loss level of *z* (i.e., chosen quantile of loss level), the function $\mathcal {J}_{j}(z, \varvec{\lambda };F)$ is greater than the function $\mathcal {J}_{j}(z, \varvec{\tau };F)$. Thus, $j=1,$ the equally weighted forecast combination $\varvec{\tau }^{^{\prime }}\widehat{\varvec{y}} _{t+h,t}$ is stochastically dominated (i.e., does not yield the optimal forecast combination) at the first order at a given quantile of loss function if some other forecast combination $\varvec{\lambda } ^{^{\prime }}\widehat{\varvec{y}}_{t+h,t}$ dominates it at a given quantile of loss level *z*. In other words, there is an alternative weighting scheme, $\varvec{\lambda }$, such that when forecasts are combined with these weights, $\varvec{\lambda }^{^{\prime }}\widehat{ \varvec{y}}_{t+h,t}$, yields a distribution of loss (i.e., distribution of forecast errors based on the loss function) that offers a lower number of forecast errors above the chosen *z* level when compared to average forecast combination.

We obtain SD at the first and second orders when $j=1$ and $j=2$, respectively. The hypothesis for testing the SDE of order *j* of the distribution of the equally weighted forecast combination $\varvec{\tau } ^{^{\prime }}\widehat{\varvec{y}}_{t+h,t}$ over the distribution of an alternative forecast combination $\varvec{\lambda }^{^{\prime }}\widehat{ \varvec{y}}_{t+h,t}$ takes analogous forms but uses a single given $\varvec{\lambda }^{^{\prime }}\widehat{\varvec{y}}_{t+h,t}$ rather than several of them.

The empirical counterpart of (2) is simply obtained by integrating with respect to the empirical distribution $\hat{F}$ of *F*, which yields the following:

3 $$\begin{aligned} \mathcal {J}_{j}(z,\varvec{\lambda };\hat{F})=\frac{1}{N_{\!f}} \sum _{N_{\!f}=1}^{N_{\!f}}\frac{1}{(j-1)!}(z-L(\varepsilon _{t+h,t}^{w}))^{j-1} \mathbb {I}\{L(\varepsilon _{t+h,t}^{w})\le z\}, \end{aligned}$$

where $N_{\!f}$ is the number of factor of realizations.^6^ In other words, $N_{\!f}$ is the number of forecasts made by different time-series models which are under evaluation. The empirical counterpart counts the number of forecast combinations that offers loss that are less than the given *z* level (i.e., given quantile of the loss distribution) when $j=1$. On the other hand, we look for the sum of the area under the integral (i.e., sum of the forecast errors) up to a given *z* level with a given forecast combination when $j=2$.

We consider the weighted Kolmogorov–Smirnov-type test statistic

4 $$\begin{aligned} \hat{S}_{j}:=\sqrt{N_{\!f}}\frac{1}{N_{\!f}}\sup _{\varvec{\lambda }}\left[ \mathcal {J}_{j}(z,\varvec{\lambda };\hat{F})-\mathcal {J}_{j}(z, \varvec{\tau };\hat{F})\right] \text { for given }z\text { level} \end{aligned}$$

and a test based on the decision rule

$$\begin{aligned} {\text {``}}\text{ Reject }\quad H_{0}^{j}\quad \text{ if }\quad \hat{S}_{j}>c_{j}\,{\text {''}}, \end{aligned}$$

where $c_{j}$ is some critical value.

To make the result operational, we need to find an appropriate critical value $c_{j}$. Because the distribution of the test statistic depends on the underlying distribution, this is not an easy task, and we decide hereafter to rely on a block bootstrap method to simulate *p*-values, where the critical values are obtained using a supremum statistic.^7^ In this context, the observations are functions of error terms that can be assumed to be serially uncorrelated. Hence, we apply the simulation methodology proposed by Barrett and Donald (2003) for i.i.d. data in multivariate context (see Barrett and Donald 2003 for details). The test statistic $\hat{S}_{1}$ for first-order stochastic dominance efficiency is derived using mixed integer programming formulations (see “Appendix”).^8^

To sum up, for a given quantile of loss distribution, we analyze whether the equally weighted forecast combination is optimal or not. We test whether an alternative combination of forecasts provides a loss distribution up to a given quantile of loss that would dominate such distribution when forecasts are combined in an equally weighted way. If an alternative combination of forecasts dominates the equally weighted combination, then there is an alternative combination which yields a distribution of loss that is the optimal one at that given quantile.

## Empirical analysis

### Data, forecasting models, and forecast methodology

In this section, we apply the SDE testing methodology to obtain optimal forecast combinations on Japanese yen/US dollar and US dollar/Great Britain pound exchange rate returns data. We use log first differences of the exchange rate levels. The exchange rate series data are expressed with a weekly frequency for the period between 1975:1 and 2010:52.^9^ The use of weekly data avoids the so-called weekend effect, as well as other biases associated with non-trading, bid-ask spread, asynchronous rates, and so on, which are often present in higher-frequency data. To initialize our parameter estimates, we use weekly data between 1975:1 and 2006:52. We then generate pseudo-out-of-sample forecasts of 2007:1–2009:52 to analyze the forecast performance at the 2007/2009 financial crisis period. We also generate pseudo- out-of-sample forecasts for the period between 2010:1 and 2012:52 to analyze the performance of the forecasts out-of-financial crisis period. Parameter estimates are updated recursively by expanding the estimation window by one observation forward, thereby reducing the pseudo-out-of-sample test window by one period.

In our out-of-sample forecasting exercise, we concentrate exclusively on univariate models, and we consider three types of linear univariate models and four types of nonlinear univariate models. The linear models are random walk (RW), autoregressive (AR), and autoregressive moving-average (ARMA) models; the nonlinear ones are logistic smooth transition autoregressive (LSTAR), self-exciting threshold autoregressive (SETAR), Markov-switching autoregressive (MS-AR), and autoregressive neural network (ARNN) models.

Let $\hat{y}_{t+h,t}$ be the forecast of $y_{t+h}$ that is generated at time *t* for the time $t+h$ ($h\ge 1$) by any forecasting model. In the RW model, $\hat{y}_{t+h,t}$ is equal to the value of $y_{t}$ at time *t*.

The ARMA model is

5 $$\begin{aligned} y_{t}=\alpha +\sum _{i=1}^{p}\phi _{1,i}y_{t-i}+\sum _{i=1}^{q}\phi _{2,i}\varepsilon _{t-i}+\varepsilon _{t}, \end{aligned}$$

where *p* and *q* are selected to minimize the Akaike information criterion (AIC) with a maximum lag of 24. After estimating the parameters of Eq. (5), one can easily produce *h*-step ($h\ge 1$) forecasts through the following recursive equation:

6 $$\begin{aligned} \hat{y}_{t+h,t}=\alpha +\sum _{i=1}^{p}\hat{\phi }_{1,i}{y}_{t+h-i}+ \sum _{i=1}^{q}\hat{\phi }_{2,i}{\varepsilon }_{t+h-i}\,. \end{aligned}$$

When $h>1$, to obtain forecasts, we iterate a one-period forecasting model by feeding the previous period forecasts as regressors into the model. This means that when $h>p\text { and }h>q$, $y_{t+h-i}$ is replaced by $\hat{y} _{t+h-i,t}$ and $\varepsilon _{t+h-i}$ by $\hat{\varepsilon }_{t+h-i,t}=0$.

An obvious alternative to iterating forward on a single-period model would be to tailor the forecasting model directly to the forecast horizon, i.e., to estimate the following equation by using the data up to *t*:

7 $$\begin{aligned} y_{t}=\alpha +\sum _{i=0}^{p}\phi _{1,i}y_{t-i-h}+\sum _{i=0}^{q}\phi _{2,i}\varepsilon _{t-i-h}+\varepsilon _{t}, \end{aligned}$$

for $h\ge 1$. We use the fitted values of this regression to directly produce an *h*-step ahead forecast.^10^

Because it is a special case of ARMA, the estimation and forecasts of the AR model can be obtained by simply setting $q=0$ in (5) and (7).

The LSTAR model is

8 $$\begin{aligned} y_{t}=\left( \alpha _{1}+\sum _{i=1}^{p}\phi _{1,i}\,y_{t-i}\right) +d_{t}\left( \alpha _{2}+\sum _{i=1}^{q}\phi _{2,i}\,y_{t-i}\right) +\varepsilon _{t}, \end{aligned}$$

where $d_{t}=\left( 1+\exp {\{-\gamma (y_{t-1}-c)\}}\right) ^{-1}$. Whereas $\varepsilon _{t}$ are regarded as normally distributed i.i.d. variables with zero mean, $\alpha _{1}$, $\alpha _{2}$, $\phi _{1,i}$, $\phi _{2,i}$, $\gamma$, and *c* are simultaneously estimated by maximum likelihood methods.

In the LSTAR model, the direct forecast can be obtained in the same manner as with ARMA, which is also the case for all of the subsequent nonlinear models^11^, but it is not possible to apply any iterative scheme to obtain forecasts for multiple steps in advance, as can be done in the case of linear models. This impossibility follows from the general fact that the conditional expectation of a nonlinear function is not necessarily equal to a function of that conditional expectation. In addition, one cannot iteratively derive the forecasts for the time steps $h>1$ by plugging in the previous forecasts (see, e.g., Kock and Terasvirta 2011).^12^ Therefore, we use the Monte Carlo integration scheme suggested by Lin and Granger (1994) to numerically calculate the conditional expectations, and we then produce the forecasts iteratively.

When $|\gamma |\rightarrow \infty$, the LSTAR model approaches the two-regime SETAR model, which is also included in our forecasting models. As with LSTAR and most nonlinear models forecasting with SETAR does not permit the use a simple iterative scheme to generate multiple-period forecasts. In this case, we employ a version of the normal forecasting error (NFE) method suggested by Al-Qassam and Lane (1989) to generate multistep forecasts.^13^ NFE is an explicit, form-recursive approximation for calculating higher-step forecasts under the normality assumption of error terms and has been shown by De Gooijer and De Bruin (1998) to perform with reasonable accuracy compared with numerical integration and Monte Carlo method alternatives.

The two-regime MS-AR model that we consider here is as follows:

9 $$\begin{aligned} y_{t}=\alpha _{s}+\sum _{i=1}^{p}\phi _{s,i}y_{t-i}+\varepsilon _{t}, \end{aligned}$$

where $s_{t}$ is a two-state discrete Markov chain with $S=\{1,2\}$ and $\varepsilon _{t}\sim$ i.i.d. $N(0,\sigma ^{2})$. We estimate MS-AR using the maximum likelihood expectation–maximization algorithm.

Although MS-AR models may encompass complex dynamics, point forecasting is less complicated in comparison with other nonlinear models. The *h*-step forecast from the MS-AR model is

10 $$\begin{aligned} \hat{y}_{t+h,t}= & {} P\left( s_{t+h}=1\mid y_{t},\ldots ,y_{0}\right) \left( \alpha _{s=1}+\sum _{i=1}^{p}\hat{\phi }_{s=1,i}{y}_{t+h-i}\right) \nonumber \\&+P\left( s_{t+h}=2\mid y_{t},\ldots ,y_{0}\right) \left( \alpha _{s=2}+\sum _{i=1}^{p}\hat{\phi }_{s=2,i}{y}_{t+h-i}\right) , \end{aligned}$$

where $P\left( s_{t+h}=i\mid y_{t},\ldots ,y_{0}\right)$ is the *i*th element of the column vector $\mathbf {P}^{h}\hat{\xi }_{t\mid t}$. In addition, $\hat{ \xi }_{t\mid t}$ represents the filtered probabilities vector and $\mathbf {P} ^{h}$ is the constant transition probability matrix (see Hamilton 1994). Hence, multistep forecasts can be obtained iteratively by plugging in $1,2,3,\ldots$-period forecasts that are similar to the iterative forecasting method of the AR processes.

ARNN, which is the autoregressive single-hidden-layer feed-forward neural network model^14^ suggested in Terasvirta (2006), is defined as follows:

11 $$\begin{aligned} y_{t}=\alpha +\sum _{i=1}^{p}\phi _{i}y_{t-i}+\sum _{j=1}^{h}\lambda _{j}d\left( \sum \limits _{i=1}^{p}\gamma _{i}y_{t-i}-c\right) +\varepsilon _{t}, \end{aligned}$$

where *d* is the logistic function, which is defined above as $d=\left( 1+\exp {\{-x\}}\right) ^{-1}$. In general, the estimation of an ARNN model may be computationally challenging. Here, we follow the QuickNet method, which is a type of “relaxed greedy algorithm”; it was originally suggested by White (2006). In contrast, the forecasting procedure for ARNN is identical to the procedure for LSTAR.

To obtain pseudo-out-of-sample forecasts for a given horizon *h*, the models are estimated by running regressions with data that were collected no later than the date $$t_{0}<T$$, where $$t_{0}$$ refers to the date when the estimation is initialized and *T* refers to the final date in our data. The first *h*-horizon forecast is obtained using the coefficient estimates from the initial regression. Next, after moving forward by one period, the procedure is repeated. For each *h*-step forecast, we calculate $$N_{\!f}$$ ($$ =T-t_{0}-h-1$$) forecast errors for each of the models that we use in our applications.

### Forecast selection and combination

Before proceeding with our application, in this section we offer different set of model selection and combination methods that are employed extensively in the literature. Akaike’s information criterion (AIC) and Bayesian information criterion (BIC) are two of the most commonly used selection criteria that serve to select a forecasting model (see, e.g., Swanson and Zeng 2001; Drechsel and Maurin 2010, among many others). The model that provides the lowest AIC or BIC, calculated as below, for a model *m* is chosen as the preferred model.

12 $$\begin{aligned} AIC(m)= & {} n\ln (\widehat{\sigma }_{m}^{2})+2k_{m}, \end{aligned}$$

13 $$\begin{aligned} BIC(m)= & {} n\ln (\widehat{\sigma }_{m}^{2})+k_{m}\ln n, \end{aligned}$$

where $\widehat{\sigma }_{m}^{2}$ is the forecast error variance estimate and $k_{m}$ is the number of regressors used in each respective model. This procedure requires the selection of the forecasting model that offers the minimum value of AIC or BIC. Another classical method that is used to select the best individual forecasting model is to select the model that offers the least forecast variance, also called predictive least squares (PLS) (Rissanen 1986).

However, these procedures neglect the fact that, as is discussed above, the combination of different models could perform better than the selection of a single model as the best model. Therefore, the procedure can be modified accordingly so that weights given to each model are determined based on the distance between each model’s *AIC* (*BIC*) from the minimal performing model’s *AIC* (*BIC*) level. Hence, defining the difference between the *AIC*(*m*) (*BIC*(*m*)) and the *min*(*AIC*) (i.e., the model that offers the lowest *AIC*) as $\Delta AIC(m)=AIC(m)-\min (AIC)$ ($\Delta BIC(m)=BIC(m)-\min (BIC)$), the exponential “Akaike weights,” $\varvec{w}_{AIC}(m)$, (see, e.g., Burnham and Anderson 2002) and “Bayesian weights,” $\varvec{w}_{BIC}(m)$ (see, e.g., Raftery 1995; Fernández et al. 2001; Sala-i-Martin et al. 2004, among many others) can be obtained as follows:

14 $$\begin{aligned} \varvec{w}_{AIC}(m)= & {} \frac{\exp \left( -\frac{1}{2}\Delta AIC(m)\right) }{ \sum _{j=1}^{M}\exp \left( -\frac{1}{2}\Delta AIC(j)\right) }, \end{aligned}$$

15 $$\begin{aligned} \varvec{w}_{BIC}(m)= & {} \frac{\exp \left( -\frac{1}{2}\Delta BIC(m)\right) }{ \sum _{j=1}^{M}\exp \left( -\frac{1}{2}\Delta BIC(j)\right) }. \end{aligned}$$

Then, these weights can be utilized to combine the forecasts of *m* models. Another commonly used method to combine forecasts is to allocate weights to each model inversely proportional to the estimated forecast error variances (Bates and Granger 1969), whereas Granger and Ramanathan (1984) employs ordinary least squares (minimizing the sum of squared errors) to obtain optimal weights for the point forecasts of individual models. Given that we also compare the distribution of loss at a given quantile of equally weighted forecasts, we also compare our findings with the weights obtained the standard quantile regression weights (Koenker 2005).

Among all these model selection and combination methods, the recent literature, as mentioned earlier, also employs the equally weighted forecast combination and the median forecast (see e.g., Stock and Watson (2004); Kolassa (2011)). All forecast model selection and combination methods discussed in this section will be employed and compared to the method with SDE weights proposed in this paper.

## Results for the efficiency of forecast combinations

This section presents our findings of the tests for first-order SD efficiency of the equally weighted forecast combination. We find that the equally weighted forecast combination is not the optimal forecast combination at all quantiles of the forecast error distribution, but it offers to be equally well in some quantiles of the distribution. It might seem that the SDE methodology finds an optimal forecast combination when compared to the equally weighted forecast combination scenario alone and ignores the performance of the rest of the available combinations. However, this is not the case. The SDE methodology finds the optimal combination from the set of all possible combinations (i.e., full diversification is allowed across different univariate forecasts). Hence, the optimal SDE forecast combination would also dominate the rest of the possible combinations as these are part of the available choice set. We obtain the best forecast combinations of the model-based forecasts for the Japanese yen/US dollar and the US dollar/Great Britain pound exchange rate forecasts by computing the weighting scheme on each forecast model that offers the optimal forecast combination at different quantiles of the loss distribution.

In our applications, because the loss distribution (i.e., absolute forecast error distribution) with the equally weighted forecast combination is known, we can obtain the number of forecast combinations that generate loss that are less than each given level of loss, *z*. In other words, one could obtain the number of forecasts that generate loss that is below a given quantile of the loss distribution with the equally weighted forecast combination. We test different quantiles of the empirical loss distribution of the average forecast combination, that is, we test whether the equally weighted forecast combination is the best forecast combination against the alternative combination at different parts of the empirical distribution. In the next section, we report the optimal forecast combination for different percentiles (i.e., 50th, 75th, 95th percentiles) of the empirical loss distribution for the two applications for different forecast periods and horizons.^15^ We also report the average of the optimal forecast combinations that are obtained for different loss levels (i.e., different quantiles of the loss distribution).^16^ For each application, we also compare the best forecast combinations obtained with SDE weights with different set of model selection and combinations that are used commonly in the literature.

### The Japanese yen/US dollar exchange rate application

First, we begin our empirical analysis with the weekly Japanese yen/US dollar exchange rate forecasts for different forecast horizons for the financial crisis period of 2007/2009 (i.e., 2007:01 and 2009:52). We proceed with testing whether the equally weighted forecast combination of the forecasting models for different horizons is the optimal forecast combination at different levels of loss or there are alternative weights on the forecast models that stochastically dominate the equally weighted forecast combination, $\varvec{\tau }^{^{\prime }}\widehat{\varvec{y} }_{t+h,t}$, in the first-order sense for some or all levels of loss, where the number of forecast combinations that generates loss above a given *z* level is minimized.^17^

Table 1 presents the results for the 50th, 75th, and 95th percentiles of the loss distribution of the equally weighted forecast combination for the different forecast horizons (*h*). The second column gives the details of the forecast period, whereas the third column reports the loss levels (i.e., absolute forecast errors) with the equally weighted forecast combinations at these particular percentiles. The following columns provide the weights of the underlying forecasting models for the optimal forecast combinations at the 50th, 75th, and 95th percentiles of the loss distribution with the equally weighted forecast combination.

**Table 1:** Optimal forecast combinations (Japanese yen/US dollar exchange rates)

Forecast horizon	Forecast period	Percentile	Forecast error	WEIGHTS						
				AR	ARMA	LSTAR	MS-AR	ARNN	RW	SETAR
$h=1$ (1 week)	2007:01–2009:12	50th	0.0109	0.0433	0.0404	0.0000	0.0000	0.0000	0.0000	0.9163
		75th	0.0181	0.9420	0.0000	0.0000	0.0000	0.0000	0.0062	0.0518
		95th	0.0364	0.8664	0.0000	0.0000	0.0000	0.0000	0.0187	0.1150
$h=26$ (6 months)	2007:07–2009:12	50th	0.0117	0.6638	0.0000	0.1542	0.0000	0.0000	0.1821	0.0000
		75th	0.0191	0.8817	0.0000	0.0000	0.0000	0.0000	0.0041	0.1142
		95th	0.0356	0.1256	0.4588	0.0000	0.0000	0.0000	0.1453	0.2703
$h=52$ (1 year)	2008:01–2009:12	50th	0.0127	0.1321	0.8679	0.0000	0.0000	0.0000	0.0000	0.0000
		75th	0.0200	0.8175	0.0000	0.0000	0.0000	0.0000	0.0977	0.0848
		95th	0.0327	0.8601	0.0000	0.0000	0.0000	0.0000	0.1399	0.0000

In one step ahead forecast horizon, i.e., when $h=1$, we have 156 forecasts for each of the different time-series models. As indicated in the first panel of Table 1, there is always an alternative forecast combination that generates less number of loss above a given loss level at the 50th, 75th, and 95th percentiles of the loss distribution (i.e., optimal forecast combination). For example, at the 50th percentile of the loss distribution, when forecasts from AR, ARMA, and SETAR obtain weights of 4.33, 4.04 and 91.63%, respectively, this combination offers the optimal combination for this part of the distribution. For the 75th percentile of the loss distribution, when forecasts from AR, RW, and SETAR obtain weights of 94.20, 0.62, and 5.18%, respectively, this combination offers the optimal combination up to this percentile. Similar to the 75th percentile of the loss distribution, AR, RW and SETAR contributes to the optimal forecast combination for the 95th percentile of the loss distribution with weights of 86.64, 1.87, and 11.50%, respectively. Overall, when $h=1$, different forecast combinations generate the best forecast combinations for different sections of the loss distribution. SETAR contributes the most to the optimal forecast combination at the 50th percentile of the loss distribution and AR contributes the most at the 75th and 95th percentiles of the loss distribution.

We carried out the same application when we extended the forecast horizon for 6 months (26 weeks) and a year (52 weeks) (i.e., $h=26$ and 52, respectively), where for each case, each model produces 130 and 104 forecasts, respectively.

For $h=26$, at the 50th and 75th percentiles, AR model contributes relatively more to the optimal forecast combination, whereas at the 95th percentile, ARMA contributes to the optimal forecast combination the most with 45.88%, followed by the contribution of the SETAR, RW, and AR models with weights of 27.03, 14.53, and 12.56%, respectively. The similar trend for the optimal forecast combination continues for $h=52$ where ARMA model contributes the most at the 50th percentile and AR model contributes the most at the 75th and 95th percentiles.

**Fig. 1:**
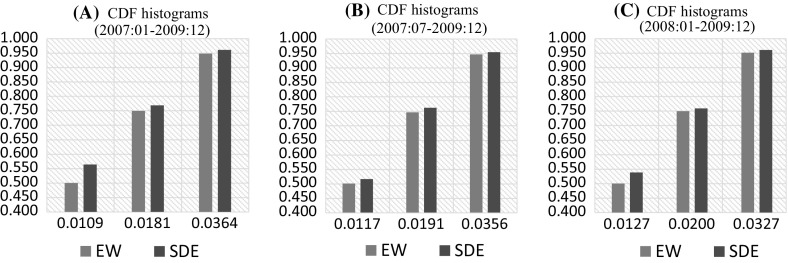
Cumulative distribution functions with the average and SDE forecast combinations for Japanese yen/US dollar exchange rate

Figure 1 shows the cumulative distribution functions of the absolute error terms with equally weighted (EW) and SDE forecast combinations for forecast periods of 2007:01–2009:12, 2007:07–2009:12 and 2008:01–2009:12 ($h=1$, 26 , and 52, respectively). Vertical and horizontal axes describe the probability and forecast error levels. For a given error level, there is always a higher portion of forecasts that offer absolute error that is below this error level with the SDE forecast combination when compared to the EW combination. In Panel A (where the forecast period is 2007:01–2009:12), 50% of the EW forecast combinations offer an error that is below 0.0117, whereas the 56.5% of the forecast combinations with SDE weights have an error that is less than this error level. One could interpret the results as follows. If a company guarantees to provide compensation to their customers if their forecasts give an error level (loss) above 0.0117, then the company would compensate 50% of its customers relying on the EW forecast combination, whereas this compensation rate would have been only 43.5% if the SDE weights would have been used.

In this subsection, we presented the best forecast combinations at different percentiles of loss distribution when we consider the equally weighted forecast combination as the “benchmark.” In the next subsection, we offer a comparison of SDE weights not only with equally weighted forecast combination but also with median forecast, model selection methods (i.e., AIC, BIC, and PLS), and the forecast combination methods (i.e., combination of forecasts with Bates and Granger, Granger and Ramanathan, AIC, and BIC weights, quantile regression).

### Comparisons

SDE weights obtained in the previous section suggested that when the equally weighted forecast combination is the benchmark, there is always an alternative forecast combination which would constitute a better case at different quantiles of the loss distribution for all forecast horizons. To evaluate SDE weights further, we also obtain median forecast, and forecasts with different model selection and combination methods that are mentioned above.

To make the results more apparent for each forecast horizon, Table 2 presents the number of forecasts with different forecast selection and combination methods that offer loss levels that are equal to or less than a given level of loss, *z*, at the 50th, 75th, and 95th percentiles with the equally weighted forecast combination (EW), median forecast (Median), forecasts with the best model chosen with AIC, BIC, and PLS, and forecast combinations with Bates and Granger, Granger and Ramanathan, AIC, BIC, and quantile regression weights.

**Table 2:** Number of forecast errors below a given forecast error (Japanese yen/US dollar exchange rates)

Forecast horizon	Forecast period	Percentile	Forecast error	Mean	Median	AIC	BIC	PLS	AIC weights	BIC weights	Bates–Granger weights	Granger–Ramanathan weights	Quantile regression weights	SDE Best
$h=1$ (1 week)	2007:01–2009:12	50th	0.0109	78	73	73	73	72	73	73	77	69	72	**88**
		75th	0.0181	117	**120**	119	119	119	119	119	118	119	117	**120**
		95th	0.0364	148	148	148	148	149	148	148	**150**	149	149	**150**
$h=26$ (6 months)	2007:07–2009:12	50th	0.0117	65	57	57	57	58	57	57	61	56	65	**67**
		75th	0.0191	97	98	98	98	**99**	98	98	98	98	95	**99**
		95th	0.0356	123	123	123	123	123	123	123	123	**124**	123	**124**
$h=52$ (1 year)	2008:01–2009:12	50th	0.0127	52	49	49	49	47	50	50	50	51	**56**	**56**
		75th	0.0200	78	78	78	78	77	78	78	78	76	78	**79**
		95th	0.0327	99	96	96	96	96	96	96	97	97	98	**100**

Bold values identify the forecast selection and/or combination model(s) that perform(s) the best at respective percentile of the forecast error distribution

In Table 2, we calculate the number of forecasts with different forecast selection and combination methods that offer loss levels that are equal to or less than a given level of loss, *z*, at the 50th, 75th, and 95th percentiles of the loss distribution from the equally weighted forecast combination. The optimal forecast combinations with the SDE weights are obtained using the weights from Table 1. Moreover, we obtain median forecast, forecasts from the model that is chosen with the AIC, BIC, and PLS criteria, and forecast combinations with Bates and Granger, Granger and Ramanathan, AIC, BIC weights, and quantile regression weights for a given percentile. Each of these methods yields loss distributions which are compared with the distribution of loss obtained with the optimal forecast combinations using the SDE weights. For example, for $h=1,$ at 50th percentile of loss distribution, there are 78 combined forecasts that generate loss levels that are less than or equal to the loss level of 0.0109 when forecasts are combined with equal weights. On the other hand, the best forecast combination with SDE weights yields 88 combined forecasts that generate loss levels that are equal to or less than 0.0109, whereas the forecasts obtained with other forecast selection and combination methods generate less number of loss levels that are equal to or less than 0.0109, suggesting that these methods offer more forecasts that give a loss level that is above 0.0109 when compared to the best case with the SDE weights. In other words, the SDE weights offer the least number of forecasts with a loss above a given threshold (which is 0.0109 in this case). If a company agrees to compensate consumers if their forecast errors are above 0.0109, then if it uses the forecast combination with SDE weights, it would need to compensate 10 less cases than the second best case offering the lowest number of forecasts above 0.0109, which in this case is the equally weighted forecast combination. Similarly, for the 75th and 95th percentiles, the best forecast combination with SDE weights performs better than the most of other forecast selection and combination methods where there are 120 and 150 forecasts that produce loss levels that are equal to or less than 0.0181 and 0.0364, respectively. In other words, the optimal forecast combinations with SDE weights produce 36 and 6 forecasts that give loss levels that are above 0.0172 and 0.0318, respectively. We also find that the median forecast and forecast combination with the Bates and Granger weights produce equally well outcomes at the 75th and 95th percentiles, respectively. However, the SDE weights offer the best or equally well position for different parts of the absolute error distribution, whereas the forecast selection and combination methods only work equally well in certain percentiles of the loss distribution.

We carry out the same analysis when we change the forecast horizons. When $$ h=26$$, at the 50th percentile of the loss distribution, SDE weights offers the least number of forecasts that give an error level above 0.0117 when compared to other methods. On the other hand, at the 75th and 95th percentiles of the loss distribution, the forecasts with PLS and forecast combination with Granger and Ramanathan weights offer an equally well, respectively. For $$h=52$$, at the 50th percentile of the loss distribution, forecast combination with quantile regression offers equally well case compared to forecast combination with SDE weights. However, at the 75th and 95th percentiles of the loss distribution, forecast combination with SDE weights offers the least number of forecasts that give an error level that is above a given level.

We only presented the SDE weights for the best forecast combination at 50th, 75th, and 95th percentiles of the loss distribution. However, Table 3 illustrates the average contribution of each forecasting model to the best forecast combination with SDE weights. These average contributions are calculated by averaging the different weights over all percentiles of the entire loss distribution. One can see that each model contributes slightly to the optimal forecast combination in different areas of the loss distribution for different forecast horizons. However, the main contributor to the optimal forecast combination is the AR model, followed by SETAR, LSTAR, and ARMA, on average considering all horizons.

**Table 3:** Average weights of optimal forecast combinations for the whole distribution (Japanese yen/US dollar exchange rates)

Forecast horizon	Forecast period	AR	ARMA	LSTAR	MS-AR	ARNN	RW	SETAR
$h=1$ (1 week)	2007:01–2009:12	0.5222	0.0253	0.0004	0.0887	0.0000	0.0119	0.3514
$h=26$ (6 months)	2007:07–2009:12	0.4491	0.1382	0.1679	0.0120	0.0074	0.0389	0.1865
$h=52$ (1 year)	2008:01–2009:12	0.4848	0.0973	0.1248	0.0000	0.0025	0.0676	0.2230

Overall, for the weekly Japanese yen/US dollar exchange rate forecasts, we find that the best forecast combination with SDE weights mostly outperforms the other forecast selection and combination models, with some few exceptions where some other models perform equally well. We also should note that the objective of the SDE weight allocation is to obtain the lowest number of forecasts that give a loss above a given threshold, not to minimize the overall loss. Hence, we do not produce conventional comparisons of different methods, but we simply compare whether SDE approach dominates other forecast selection and combination methods given the loss level. For example, when $h=1$, if one were to use conventional comparisons, for the 50th percentile, the combination obtained with the quantile regression offers the lowest mean absolute error for this percentile compared to other methods. In other words, if the forecaster’s objective is to minimize the aggregate (or mean) loss up to a given forecast percentile, the forecast combination through quantile regression would be a better model to use. Yet, if the forecaster’s objective is to minimize the number of forecasts that gives a loss above a given level, then SDE weights offer better (and in a few cases equally well) forecast combinations compared to any other forecast selection and combination. Therefore, forecast combinations with the SDE methodology offer a complementary approach to the standard forecast selection/combination methods used in the forecasting literature as they can produce better outcomes if one were to minimize the number of forecasts with a loss above a given threshold.

### US dollar/Great Britain pound exchange rate application

In this subsection, we obtain the optimal forecast combination for the foreign exchange rate of US dollar/Great Britain pound forecasts for different time horizons at different quantiles of the loss distribution for the financial crisis period of 2007/2009 (i.e., 2007:01 and 2009:52). Table 4 presents the best forecast combinations with SDE method at the 50th, 75th, and 95th percentiles of the loss distribution of the equally weighted forecast combination when $h=1$, 26, and 52, respectively. Table 5 reports the number of forecasts with different forecast selection and combination methods that offer loss levels that are equal to or less than a given level of loss for different percentiles of the loss distribution. Finally, Table 6 presents the average SDE weights of each model that contributes to the optimal forecast combination.

**Table 4:** Optimal forecast combinations (US dollar/Great Britain pound exchange rates)

Forecast horizon	Forecast period	Percentile	Forecast error	WEIGHTS						
				AR	ARMA	LSTAR	MS-AR	ARNN	RW	SETAR
$h=1$ (1 week)	2007:01–2009:12	50th	0.0100	0.0000	0.3567	0.0000	0.0000	0.4825	0.0000	0.1608
		75th	0.0193	0.6490	0.0000	0.0000	0.0000	0.0000	0.1139	0.2371
		95th	0.0430	0.4822	0.0000	0.0000	0.0000	0.4852	0.0326	0.0000
$h=26$ (6 months)	2007:07–2009:12	50th	0.0125	0.6431	0.0000	0.0000	0.0000	0.0000	0.0028	0.3541
		75th	0.0215	0.6275	0.3726	0.0000	0.0000	0.0000	0.0000	0.0000
		95th	0.0410	0.5297	0.0000	0.0000	0.0000	0.0000	0.2628	0.2075
$h=52$ (1 year)	2008:01–2009:12	50th	0.0121	0.0392	0.4499	0.0000	0.0000	0.0000	0.0687	0.4422
		75th	0.0235	0.8430	0.0000	0.0000	0.0000	0.0000	0.1570	0.0000
		95th	0.0433	0.8677	0.0000	0.0000	0.0000	0.0000	0.0100	0.1223

The optimal weights obtained for the foreign exchange rate of US dollar/Great Britain pound are very similar to the ones obtained for the Japanese yen/US dollar exchange rate data (see Table 4 for details). For $$ h=1$$, AR, ARMA, ARNN  and SETAR are the main contributors to the optimal forecast combination with SDE weights with differing levels of contribution in different percentiles. AR model contributes the most to the optimal forecast combination at 50th, 75th, and 95th percentiles of the loss distribution when $$h=26$$. Finally, when $$h=52$$, ARMA and SETAR contribute the most to the optimal forecast combination at the 50th percentile and AR model is the main contributor to the optimal forecast combination at the 75th and 95th percentiles.

**Fig. 2:**
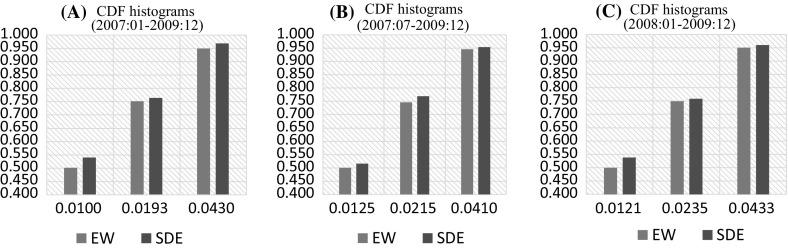
Cumulative distribution functions with the EW and SDE forecast combinations for US dollar/British pound exchange rate

Figure 2 shows the cumulative distribution functions of the absolute error terms with equally weighted (EW) and SDE forecast combinations for forecast periods of 2007:01–2009:12, 2007:07–2009:12 and 2008:01–2009:12 ($h=1$, 26, and 52, respectively). Vertical and horizontal axes offer the probability and forecast error levels. For a given error level, there is always a higher portion of forecasts that produce absolute errors below this level with the SDE forecast combination when compared to the EW combination. In Panel A (where the forecast period is 2007:01–2009:12), 50% of the EW forecast combinations offer an error that is below 0.01, whereas the 54% of the forecast combinations with SDE weights have an error that is less than this error level.

**Table 5:** Number of forecast errors below a given forecast error (US dollar/Great Britain pound exchange rates)

Forecast horizon	Forecast period	Percentile	Forecast error	Mean	Median	AIC	BIC	PLS	AIC weights	BIC weights	Bates–Granger weights	Granger–Ramanathan weights	Quantile regression weights	SDE Best
$h=1$ (1 week)	2007:01–2009:12	50th	0.0100	78	82	82	82	82	82	82	80	81	**84**	**84**
		75th	0.0193	117	117	117	117	118	117	117	117	**119**	**119**	**119**
		95th	0.0430	148	**151**	**151**	**151**	148	**151**	**151**	148	147	148	**151**
$h=26$ (6 months)	2007:07–2009:12	50th	0.0125	65	65	65	65	65	64	64	64	62	**67**	**67**
		75th	0.0215	97	99	99	99	97	99	99	96	98	99	**100**
		95th	0.0410	123	121	121	121	122	121	121	122	121	123	**124**
$h=52$ (1 year)	2008:01–2009:12	50th	0.0121	52	54	54	54	53	54	54	53	54	55	**56**
		75th	0.0235	78	76	76	76	78	76	76	77	77	78	**79**
		95th	0.0433	99	**100**	**100**	**100**	97	**100**	**100**	99	95	97	**100**

Bold values identify the forecast selection and/or combination model(s) that perform(s) the best at respective percentile of the forecast error distribution

Table 5 summarizes the comparisons of performance of different models at different sections of the loss distribution for different horizons. SDE weights for the best forecast combination outperform the other forecast selection and combination models for $h=26$ at 75th and 95th percentiles of the loss distribution. Similarly, when $h=52$, forecast combination with the SDE weights outperforms the other forecast selection and combination models at the 50th and 75th percentiles of the loss distribution. However, when $h=1$, at 50th, 75th, and 95th percentiles, there are always other forecast selection and/or combination methods that perform equally well. These cases are obtained by the forecast combination with quantile regression at the 50th percentile; forecast combinations obtained by the Granger and Ramanathan and quantile regression weights at the 75th percentile; and forecasts obtained with the median, AIC and BIC methods and forecast combinations with the AIC and BIC weights. Overall, we find that the best forecast combination with SDE weight performs better than other forecast selection and combination cases in most of the cases with very few cases where other forecast selection and combination methods offer equally well outcomes.

**Table 6:** Average weights of optimal forecast combinations for the whole distribution (US dollar/Great Britain pound exchange rates)

Forecast horizon	Forecast period	AR	ARMA	LSTAR	MS-AR	ARNN	RW	SETAR
$h=1$ (1 week)	2007:01–2009:12	0.3182	0.1317	0.0000	0.0598	0.2984	0.0228	0.1691
$h=26$ (6 months)	2007:07–2009:12	0.6070	0.0875	0.0201	0.0722	0.0007	0.0269	0.1857
$h=52$ (1 year)	2008:01–2009:12	0.4848	0.0973	0.1248	0.0000	0.0025	0.0676	0.2230

On average, forecasts from the AR, SETAR, ARMA, and ARNN models contribute the most to the optimal forecast combination obtained with SDE weights with different contribution levels at different forecast horizons (see Table 6 for details). However, these models contribute differently at different parts of the loss distribution. For example, the AR model contributes the most to the optimal forecast combination when at the 75th and 95th percentiles of the loss distribution for all horizons considered, whereas forecasts from the ARMA model contributes relatively more to the optimal forecast combination at the 50th percentile of the loss distribution for $h=1$ and $h=52$. Overall, the AR model is the main contributor to the optimal forecast combination throughout the error distribution, and SETAR, ARMA, and ARNN models contribute significantly more to the optimal forecast combination at different horizons and percentiles (see Tables 4, 6 for details).

## Robustness analysis

### Different forecast periods and out-of-sample performance

In the previous section, we considered the financial period (i.e., forecasts obtained between 2007 and 2009) and we find that the forecast combinations obtained with the SDE produce the lowest number of forecasts that give a loss above a given threshold in most of the cases analyzed over this period. In this section, we repeat our analysis to obtain optimal forecast combination for the US dollar/Great Britain pound and Japanese yen/US dollar exchange rate forecasts with the SDE methodology with the forecasts obtained for the period between 2010 and 2012 and compare its performance with other forecast selection and combination methods.

**Table 7:** Optimal forecast combinations with the forecasts between 2010 and 2012

Forecast horizon	Forecast period	Percentile	Forecast error	WEIGHTS						
				AR	ARMA	LSTAR	MS-AR	ARNN	RW	SETAR
Panel A: Forecast combinations for the Japanese yen/US dollar exchange rates										
$h=1$ (1 week)	2010:01–2012:12	50th	0.0079	0.9658	0.0000	0.0000	0.0000	0.0000	0.0000	0.0342
		75th	0.0138	0.7616	0.0000	0.0000	0.0000	0.0000	0.1534	0.0850
		95th	0.0249	0.0152	0.0000	0.0000	0.0000	0.0000	0.0000	0.9848
$h=26$ (6 months)	2010:07–2012:12	50th	0.0078	0.2703	0.0000	0.0000	0.0000	0.0000	0.1201	0.6096
		75th	0.0134	0.1688	0.0000	0.0000	0.0213	0.0000	0.1171	0.6928
		95th	0.0235	0.4868	0.0000	0.3840	0.0000	0.0000	0.1292	0.0000
$h=52$ (1 year)	2011:01–2012:12	50th	0.0077	0.9849	0.0000	0.0000	0.0000	0.0000	0.0151	0.0000
		75th	0.0125	0.6097	0.0000	0.0000	0.0000	0.0000	0.0895	0.3008
		95th	0.0270	0.1500	0.0000	0.8000	0.0000	0.0000	0.0000	0.0500
Panel B: Forecast combinations for the US dollar/Great Britain pound exchange rates										
$h=1$ (1 week)	2010:01–2012:12	50th	0.0091	0.4338	0.0000	0.0000	0.0000	0.0000	0.0210	0.5453
		75th	0.0143	0.0087	0.0000	0.0000	0.0000	0.0000	0.0535	0.9379
		95th	0.0193	0.5766	0.0000	0.0000	0.0000	0.0000	0.0830	0.3404
$h=26$ (6 months)	2010:07–2012:12	50th	0.0082	0.9333	0.0000	0.0000	0.0000	0.0000	0.0029	0.0639
		75th	0.0124	0.0917	0.0000	0.0000	0.0000	0.0000	0.0000	0.9083
		95th	0.0191	0.1651	0.0000	0.0000	0.0000	0.0000	0.0000	0.8349
$h=52$ (1 year)	2011:01–2012:12	50th	0.0080	0.4293	0.0000	0.5628	0.0000	0.0000	0.0079	0.0000
		75th	0.0128	0.9294	0.0000	0.0000	0.0000	0.0000	0.0056	0.0650
		95th	0.0190	0.2762	0.0000	0.0000	0.0000	0.0000	0.0000	0.7238

Panels A and B of Table 7 summarize the forecast combinations obtained with the SDE methodology for the Japanese yen/US dollar and US dollar/Great Britain pound exchange rate forecasts, respectively. Absolute forecast errors obtained with the equally weighted forecast combination are given at the 50th, 75th, and 95th percentiles when different forecast horizons are used. When compared to the financial crisis period (see Tables 1, 4 forecast error levels at different percentiles), after the financial crisis, the equally weighted combination produced better forecasts at all horizons. However, the SDE methodology produced an alternative forecast combination that dominated the equally weighted one at a given level. For the Japanese yen/US dollar (Panel A of Table 7), we find that AR model contributes the most to the forecast combination at the 50th and 75th percentiles of the loss distribution for   $$h=1$$ and $$h=52$$, and 95th percentile of the loss distribution for $$h=26$$, whereas the SETAR model contributes the most to the optimal forecast combination at the 95th percentile of the loss distribution for $$h=1$$ and at the 50th and 75th percentiles of the loss distribution for $$h=26$$. Finally, LSTAR is the other model that contributes significantly high to the optimal combination at the 95th percentiles of the loss distribution for $$h=26$$ and $$ h=52$$. On the other hand, with the US dollar/Great Britain pound exchange rate application, the AR model contributes significantly high levels to the optimal combination at the 50th and 75th percentiles of the loss distribution for $$h=1$$, at the 50th percentile of the loss distribution for $$h=26$$, 50th and 75th percentiles of the loss distribution for $$h=52$$. Similarly, SETAR is the other model that contributes significantly to the optimal forecast combination at the 50th, 75th, and 95th percentiles for $$h=1$$, at the 75th and 95th percentiles of the loss distribution for $$h=26$$, and 95th percentile of the loss distribution for $$h=52$$. Finally, the LSTAR model contributes the most to the optimal combination at the 50th percentile of the loss distribution for $$h=52$$. Overall, for the 50th, 75th, and 95th percentiles of the loss distributions with different forecast horizons, AR and SETAR models are the main ones that contribute significantly to the optimal forecast combination, where LSTAR also contributes significantly in few cases, whereas other models’ contributions are either minimal or none.

**Table 8:** Distribution of forecasts errors with the forecast combination/selection methods in the period between 2010 and 2012

Forecast horizon	Forecast period	Percentile	Forecast error	Mean	Median	AIC	BIC	PLS	AIC weights	BIC weights	Bates–Granger weights	Granger–Ramanathan weights	Quantile regression weights	SDE Best
Panel A: Number of forecast errors below a given forecast error (Japanese yen/US dollar exchange rates)														
$h=1$ (1 week)	2010:01–2012:12	50th	0.0079	78	79	79	79	78	79	79	79	78	**81**	**81**
		75th	0.0138	117	113	113	113	113	113	113	115	113	114	**121**
		95th	0.0249	148	147	147	147	147	147	147	147	147	148	**150**
$h=26$ (6 months)	2010:07-2012:12	50th	0.0078	65	**67**	**67**	**67**	**67**	**67**	**67**	66	**67**	**67**	**67**
		75th	0.0134	97	97	95	95	96	95	95	100	101	101	**102**
		95th	0.0235	123	122	122	122	121	122	122	122	119	119	**124**
$h=52$ (1 year)	2011:01–2012:12	50th	0.0077	52	55	55	55	55	55	55	52	53	**56**	**56**
		75th	0.0125	78	78	78	78	78	78	78	78	78	**79**	**79**
		95th	0.0270	99	100	100	100	100	100	100	99	99	100	**101**
Panel B: Number of forecast errors below a given forecast error (US dollar/Great Britain pound exchange rates)														
$h=1$ (1 week)	2010:01–2012:12	50th	0.0091	78	79	79	79	79	79	79	80	75	**83**	**83**
		75th	0.0143	117	122	122	122	123	122	122	122	119	124	**125**
		95th	0.0193	148	146	146	146	147	146	146	147	140	147	**149**
$h=26$ (6 months)	2010:07–2012:12	50th	0.0082	65	72	72	72	72	72	72	70	70	**73**	**73**
		75th	0.0124	97	96	96	96	96	95	95	97	95	99	**101**
		95th	0.0191	123	123	123	123	123	123	123	122	123	123	**124**
$h=52$ (1 year)	2011:01–2012:12	50th	0.0080	52	57	57	57	57	57	57	54	56	**58**	**58**
		75th	0.0128	78	83	83	83	82	83	83	82	83	83	**84**
		95th	0.0190	99	100	100	100	100	100	100	100	99	**101**	**101**

Bold values identify the forecast selection and/or combination model(s) that perform(s) the best at respective percentile of the forecast error distribution

Similar to the previous section, we provide comparisons of forecast combination obtained with the SDE methodology with the standard forecast selection and combination methods where Panels A and B of Table 8 summarize the results for the Japanese yen/US dollar and US dollar/Great Britain pound exchange rate, respectively. With few exceptional cases, the forecast combinations obtained with the SDE produce a minimum number of forecasts that have a loss above a given level. The second best model for the application at hand is the quantile regression which produces equally well outcomes in some cases. In particular, with the Japanese yen/US dollar exchange rate application, the quantile regression also produces the best case at the 50th percentiles of the loss distribution at all forecast horizons and 75th percentile of the loss distribution for $h=52$ (see Panel A of Table 8). On the other hand, with the US dollar/Great Britain pound exchange rate application, the quantile regression offers equally well results at the 50th percentiles of the loss distribution at all forecast horizons and 95th percentile of the loss distribution for $h=52$ (see Panel B of Table 8).

Tables 7 and 8 present the forecast combinations and comparisons at 50th, 75th, and 95th percentiles of the loss distribution, respectively, yet we obtain forecast combinations for all percentiles of the loss distribution. Panels A and B of Table 9 give the average contribution of each forecasting model to the best forecast combination with SDE weights for the Japanese yen/US dollar and US dollar/Great Britain pound exchange rate, respectively. On average, the AR model contributes the most to the optimal combination at all horizons, followed by the SETAR model. With respect to Japanese yen/US dollar application, the AR is the main model contributing the most at all horizons, whereas the second most contributing model is the LSTAR (SETAR) when $h=1$ ($h=26$ and $h=52$) for the forecasting period after the financial crisis. When we compare the after crisis period results with the one before the crisis (see Table 3), AR is the main model contributing to the optimal forecast combination in both cases, followed by SETAR. On the other hand, ARMA model’s contribution to the optimal forecast has decreased at all horizons. The LSTAR model’s contribution to the optimal forecast has increased for $h=1$ but decreased for $h=26$ and $h=52$. For the US dollar/Great Britain pound exchange rate application, when we compare the results with respect to the crisis period (see Table 6), AR and SETAR models are significant contributors in each case, however, both AR and SETAR models’ contribution to the optimal forecast combination is significantly higher at all horizons for the after the financial crisis period. Similar to the Japanese yen/US dollar application, the ARMA model’s contribution to the optimal combination is lower for the after the crisis period. Similarly, on average, the contributions of ARNN for $h=1$ and LSTAR for $h=52$ are lower after the crisis.

**Table 9:** Average weights of optimal forecast combinations for forecast period of 2010–2012

Forecast horizon	Forecast period	AR	ARMA	LSTAR	MS-AR	ARNN	RW	SETAR
Panel A: Japanese yen/US dollar exchange rate								
$h=1$ (1 week)	2010:01–2012:12	0.4238	0.0083	0.2968	0.0099	0.0576	0.0181	0.1854
$h=26$ (6 months)	2010:07–2012:12	0.5660	0.0000	0.1539	0.0382	0.0000	0.0277	0.2143
$h=52$ (1 year)	2011:01–2012:12	0.6325	0.0000	0.0020	0.0752	0.0037	0.0238	0.2599
Panel B: US dollar/Great Britain pound exchange rate								
$h=1$ (1 week)	2010:01–2012:12	0.6254	0.0142	0.0354	0.0266	0.0000	0.0109	0.2875
$h=26$ (6 months)	2010:07–2012:12	0.6634	0.0499	0.0135	0.0011	0.0000	0.0065	0.2656
$h=52$ (1 year)	2011:01–2012:12	0.5840	0.0196	0.0000	0.0777	0.0097	0.0110	0.2981

Overall, the SDE model still produces the optimal forecast combination even after changing the forecast period in most percentiles (with few exceptions where other model selection and combination methods produce an equally well outcomes) where there is always a lower number of forecasts that produce a loss above a given threshold. The only difference between the during and the after the financial crisis periods is that the AR and SETAR models contribute relatively more to the optimal combination after the financial crisis period, while the contributions of ARMA, ARNN, and SETAR models are relatively less after the crisis period when compared to the financial crisis period.

**Table 10:** Out-of-sample performance of forecast combination/selection methods (US dollar/Great Britain pound exchange rates)

Forecast horizon	Forecast period	Percentile	Forecast error	Mean	Median	AIC	BIC	PLS	AIC weights	BIC weights	Bates–Granger weights	Granger–Ramanathan weights	SDE Best
$h=1$ (1 week)	2013:01–2014:12	50th	0.0064	52	49	48	48	49	50	47	47	47	**53**
		75th	0.0094	78	73	73	73	73	72	73	74	74	**79**
		95th	0.0189	99	98	98	98	98	98	98	99	99	**100**

Bold values identify the forecast selection and/or combination model(s) that perform(s) the best at respective percentile of the forecast error distribution

We also evaluate the out-of-sample performance of the SDE forecast combination when compared to the out-of-sample performance of all other forecast selection and combination models. To do this, we use forecast combination weights obtained for 2010–2012 period for one-step ahead forecasts for the US dollar/Great Britain pound exchange rates (i.e., weights offered in Panel B of Table 7 for the case of $h=1$) to obtain forecasts for the 2013–2014 period (104 weekly observations). We also use the in-sample choices made for the different forecast selection and combination models to obtain forecasts for the 2013–2014 period. Table 10 presents the out-of-sample performance results of the different forecast selection and combination models. At the 50th, 75th, and 95th percentiles of the error distribution, the out-of-sample performance of the forecast combination obtained with SDE is better than those from the other forecast selection and combination models.^18^ In all cases, the forecast combination with SDE has the highest number of forecasts that give errors that are less than a given threshold error. To put it differently, the forecast combination with the SDE methodology results in the least number of forecasts with an error that is above a given threshold error level when compared to the other methods. Overall, the SDE forecast combination not only works well for in-sample but also better for out-of-sample forecasts.

### Quadratic loss function

It has been well discussed in the literature that when the objective loss function is altered, then the solutions to the optimal forecast combination also alter. In particular, if the forecast error distribution is skewed, different weighted forecast combinations would work better at different parts of the empirical distribution of the forecast errors (Elliott and Timmermann 2004). For example, replacing the quadratic loss function with the absolute loss function leads to quantile regression for the median, or in other words, least absolute deviation regression (see Nowotarski et al. 2014). Hence, the quantile regression is less sensitive to the outliers compared to the squared forecast error distribution. However, both weights obtained through quantile regression and minimizing squared forecast errors are aiming to minimize a single measure (i.e., mean absolute deviation and mean squared forecast error) and changing the loss function (i.e., squaring the absolute forecast errors in this case) will alter the optimal forecast combination since the magnitude of the loss is being altered. When the magnitude of the loss is changed, then the forecast combination that minimizes the overall aggregate measure (e.g., mean squared forecast errors vis-a-vis mean absolute forecast error) will be different. However, SDE methodology does not aim to minimize the overall loss function, but tries to minimize the number of forecasts that give loss above a given level, and the optimal forecast combination with either absolute or quadratic loss function will be the same.

Let us expand our discussion on this. SDE approach’s objective is to minimize the number of forecasts that give a loss level above a given loss level. In the previous section, SDE approach finds a weight allocation across the forecast models ($\varvec{\lambda }$) that minimize the number of absolute forecast errors above a given loss level, *z*, (i.e., given absolute forecast error level), which is obtained by the following loss function: $\left| y_{t+h}-\varvec{\lambda }^{^{\prime }} \widehat{\varvec{y}}_{t+h,t}\right|$. For example, when equally weighted forecast combination ($\varvec{\tau }^{^{\prime }}\widehat{ \varvec{y}}_{t+h,t}$) is used, one already knows the distribution of the absolute forecast errors obtained from $\left| y_{t+h}-\varvec{\tau } ^{^{\prime }}\widehat{\varvec{y}}_{t+h,t}\right|$ where absolute errors are given in ascending order, $0\le \varepsilon _{1}<\varepsilon _{2}\ldots <\varepsilon _{N}$. Given the threshold loss level (*z*), we can consider that 50% of the forecasts give absolute forecast errors that is above this level with the equally weighted forecast combination. If one were to change the loss function to obtain the distribution of the squared forecast errors: $(y_{t+h}-\varvec{\tau }^{^{\prime }}\widehat{ \varvec{y}}_{t+h,t})^{2}$, the ascending distribution of the squared errors will be the same but only squared this time, i.e., $0\le \varepsilon _{1}^{2}<\varepsilon _{2}^{2}\ldots <\varepsilon _{N}^{2}$. Now, given threshold loss level ($z^{2}$), 50% of the forecasts will give squared forecast error above this threshold. The similar logic applies when one were to find the optimal weight allocation through SDE. Hence, the optimal forecast combination obtained with either loss function will offer the same result. Clearly, if one were to minimize the absolute forecast deviation (and minimized squared forecast deviation) for all the forecasts, loss function will alter the results as the magnitude of the errors would have been different but not the order and distribution of the errors (or squared errors) at a given quantile of the loss distribution.

In the previous section, we used the absolute forecast error distribution to find the optimal forecast combination for given percentiles of the error distribution. In this section, we use the squared forecast errors to obtain optimal weights with the SDE approach for the same percentiles. We use the weekly Japanese yen/US dollar exchange rate forecasts for the financial crisis period of 2007/2009 (i.e., 2007:01 and 2009:52) with the quadratic loss function where the 50th, 75th, and 95th percentiles of the squared forecast errors for $h=1$. To provide a similar distribution of squared forecast errors when compared to the absolute forecast errors, we use higher decimal places to identify the percentiles of the squared forecast errors. As expected, optimal weights obtained with the SDE methodology are the same as the one found in Table 1 given for $h=1$. Similarly, we compare the performance of the SDE weights with different forecast selection and combination methods at the 50th, 75th, and 95th percentiles of the squared forecast errors and the results are presented in Table 11.^19^ Given the squared forecast error level, for example, 0.000118, there is always a higher proportion of forecasts that produce squared forecast errors above this threshold with the forecasts obtained with forecast selection and combination methods compared with the one obtained with the SDE weights.

**Table 11:** Number of squared forecast errors below a given squared forecast error level (Japanese yen/US dollar exchange rates)

Forecast horizon	Forecast period	Percentile	Forecast error	Mean	Median	AIC	BIC	PLS	AIC weights	BIC weights	Bates–Granger weights	Granger–Ramanathan weights	Quantile regression weights	SDE Best
$h=1$	2007:01–2009:12	50th	0.000118	78	73	73	73	72	73	73	77	69	72	**88**
		75th	0.000327	117	**120**	119	119	119	119	119	118	119	117	**120**
		95th	0.001325	148	148	148	148	149	148	148	**150**	149	149	**150**

Bold values identify the forecast selection and/or combination model(s) that perform(s) the best at respective percentile of the forecast error distribution

Overall, our findings are robust to the choice of the loss function (i.e., either absolute forecast error or squared forecast errors) as altering the loss function does not alter the order of losses obtained with different forecast selection and combination methods. Changing the loss function will indeed change the optimal forecast combination obtained by the mainstream methods used in the forecasting literature as these methods consider all forecasts and minimize the overall deviation or loss (e.g., quadratic loss function gives more weight to the large forecast errors compared to least absolute deviation). However, the SDE methodology minimizes the number of forecasts that gives a loss above a given threshold level and changing the loss function do not alter the position of the losses in the distribution and the results are robust to the choice of the loss function.

## Conclusion

In this paper, we provide SDE properties to combine forecasts by which optimal forecast combinations are obtained at different quantiles of the loss distribution when compared with respect to all possible forecast combinations constructed from a set of time-series model forecasts. The SDE approach differs from the mainstream forecast combination approaches and complements them. In particular, mainstream forecast combination methods minimize the total sum of losses (such as the sum of squared forecast errors or absolute forecast errors), but the SDE methodology obtains the forecast combinations that will minimize the number of forecasts that produce losses above a given threshold rather than the aggregate measure of loss. In that respect, the SDE approach complements the existing forecast selection and/or combination methods when the forecasting priority is to minimize the number of forecasts that produce loss levels above a given threshold. In that respect, the SDE methodology is particularly well suited for the cases when a company (such as an insurance company) promises to compensate its consumers if their losses (forecast errors) are above a threshold error level rather than trying to minimize the overall loss.

We applied the SDE methodology to construct the optimal forecast combination for different forecast horizons at different percentiles of the loss distribution for weekly Japanese yen/US dollar and US dollar/Great Britain pound foreign exchange rate forecasts during and after the financial crisis. During the financial crisis period, we find that the optimal forecast combination in different areas of the loss distribution for different forecast horizons differs. However, the main contributor to the optimal forecast combination is the AR model both during and after the financial crisis period. Overall, there is also agreement that the SETAR, LSTAR, ARMA, and ARNN models contribute more to the optimal forecast combination at some parts of the loss distribution during the crisis period. However, after the crisis period, only SETAR (the second main contributor to the optimal forecast) and LSTAR are the models that contribute to the optimal forecast and contributions of ARMA and ARNN to the optimal forecast combination after the crisis period is limited compared to the crisis period.

In summary, for the majority of the cases considered, forecast combinations with SDE weights perform better than median forecasts, forecasts from the model that is chosen with *AIC*, *BIC*, and PLS, and forecast combination with equal, Bates and Granger, Granger and Ramanathan, AIC, BIC, and quantile regression weights at different parts of the loss distribution. However, there are also few cases where some other forecast selection and combination model may perform equally well at some parts of the loss distribution. In particular, forecast combination obtained with the quantile regression is the second best way of combining forecast in most of the cases.

To test the robustness of the SDE weights, we also used the quadratic loss function in our analysis. Both the weights obtained with the SDE and the comparison results with the other methods remained the same when we used the squared forecast error distribution. In particular, the SDE methodology minimizes the number of forecasts that gives a loss above a given threshold level and changing the loss function would not alter the position of the forecast errors in the distribution and as such the results are robust to the choice of the loss function.

Finally, we only applied the SDE analysis to two specific data sets with a given number (seven) of time-series models and, as such, our results on the optimality of the forecast combination at different quantiles of loss distribution do not generalize beyond the scope of the applications at hand. However, the SDE methodology can offer a useful way of assessing the optimality of forecast combinations by using information available in the entire forecast error distribution and not merely in the first two moments, as typically assumed in the literature.

## Appendix

### Mathematical formulation of the test statistics

The test statistic $\hat{S}_{1}$ for first-order stochastic dominance efficiency is derived using mixed integer programming formulations. The following is the full formulation of the model:

16 $$\begin{aligned} \mathtt {\max _{\varvec{\lambda }}}&\hat{S}_{1}=\sqrt{N_{\!f}}\frac{1}{ N_{\!f} }\sum _{N_{\!f}=1}^{N_{\!f}}(W_{N_{\!f}}-K_{N_{\!f}})\text { for a given }z\text { level } \end{aligned}$$

17 $$\begin{aligned} \mathtt {\text{ s.t. }}&M(K_{N_{\!f}}-1)\le z-L(\varepsilon _{t+h,t}^{ew})\le MK_{N_{\!f}},\quad \quad \quad \forall \,N_{\!f} \end{aligned}$$

18 $$\begin{aligned}&M(W_{N_{\!f}}-1)\le z-L(\varepsilon _{t+h,t}^{w})\le MW_{N_{\!f}},\quad \quad \quad \forall \,N_{\!f} \end{aligned}$$

19 $$\begin{aligned}&\varvec{e}^{\prime }\varvec{\lambda }=1, \end{aligned}$$

20 $$\begin{aligned}&\varvec{\lambda }\ge 0, \end{aligned}$$

21 $$\begin{aligned}&W_{N_{\!f}}\in \{0,1\},\,K_{N_{\!f}}\in \{0,1\},\quad \quad \quad \forall \,N_{\!f} \end{aligned}$$

with *M* being a large constant.

The model is a mixed integer program maximizing the distance between the two binary variables, $\displaystyle \frac{1}{N_{\!f}} \sum \nolimits _{N_{\!f}=1}^{N_{\!f}}K_{N_{\!f}}$ and $\displaystyle \frac{1}{N_{\!f}} \sum \nolimits _{N_{\!f}=1}^{N_{\!f}}W_{N_{\!f}}$, which represent $G(z,\varvec{\tau }; \hat{F})$ and $G(z,\varvec{\lambda };\hat{F})$, respectively (the empirical cdf of the loss functions with the forecast combinations, $\varvec{\tau }^{^{\prime }}\widehat{\varvec{y}}_{t+h,t}$ and $\varvec{\lambda }^{^{\prime }}\widehat{\varvec{y}}_{t+h,t}$, respectively, at loss level of *z*). According to inequality (18), $K_{N_{\!f}}$ equals 1 for each scenario of realization factors $N_{\!f}$ for which $z\ge L(\varepsilon _{t+h,t}^{ew})$ and equals 0 otherwise. Analogously, inequality (19) ensures that $W_{N_{\!f}}$ equals 1 for each scenario for which $z\ge L(\varepsilon _{t+h,t}^{w})$. Equation (20) defines the sum of all forecast combination weights to be unity, while inequality (21) disallows for negative weights. If the distance between two binary variables is positive, this means that the number of forecast combinations producing error levels with the $\varvec{\lambda } ^{^{\prime }}\widehat{\varvec{y}}_{t+h,t}$ up to a given *z* level is greater than the $\varvec{\tau }^{^{\prime }}\widehat{\varvec{y}} _{t+h,t}$. Hence, the number of forecast combinations producing error level above a given *z* level is lower with $\varvec{\lambda }^{^{\prime }} \widehat{\varvec{y}}_{t+h,t}$ than $\varvec{\tau }^{^{\prime }} \widehat{\varvec{y}}_{t+h,t}$.

This formulation allows us to test the SD of the equally weighted forecast combination, $\varvec{\tau }^{^{\prime }}\widehat{\varvec{y}} _{t+h,t}$, over any potential linear forecast combination, $\varvec{\ \lambda }^{^{\prime }}\widehat{\varvec{y}}_{t+h,t}$, of the forecasts based on time-series models. When some of the variables are binary, corresponding to mixed integer programming, the problem becomes non-polynomial (NP)-complete (i.e., formally intractable). The problem can be reformulated to reduce the solving time and to obtain a tractable formulation (see Sect. 4.1 of ST, for the derivation of this formulation and details on its practical implementation).

## Footnotes

1
Smith and Wallis (2009) found that the finite sample error is the reason behind the forecast combination puzzle. Aiolfi et al. (2011) suggested that potential improvements can be made by using a simple equally weighted average of forecasts from various time-series models and survey forecasts. See also Diebold and Pauly (1987), Clements and Hendry (1998, 1999, 2006), and Timmermann (2006) for a discussion of model instability and Elliott and Timmermann (2005) forecast combinations for time-varying data.

2 In a related paper, Pinar et al. (2013) used a similar approach to construct an optimal Human Development Index (HDI). See also Pinar et al. (2015) for optimal HDI for MENA region, Pinar (2015) for optimal governance indices, and Agliardi et al. (2015) for environmental index. The same methodology was applied in Agliardi et al. (2012), where an optimal country risk index was constructed following SD analysis with differential component weights, yielding an optimal hybrid index for economic, political, and financial risk indices that do not rely on arbitrary weights as rating institutions do (see also Agliardi et al. 2014 for Eurozone case).

3 In this paper, loss function is based on the magnitude of the forecast errors. Hence, we take the absolute values of negative errors and evaluate the errors based on their magnitude, that is, the distance from zero error, not the sign of errors.

4 As suggested by the assumptions above, we concentrate on the magnitude of the forecast errors, and therefore, *z* represents the monotonic non-decreasing distance to zero error. Throughout the paper, we refer to *z* as “loss” level so this could be clearly identified as magnitude of the forecast error rather than forecast error itself.

5 In general, combination with $\tau$ will be considered as dominating one when $G(z,\tau ;F)$ lays below the $G(z,\lambda ;F)$ when the dominant combination refers to a “best outcome” case because there is more mass to the right of *z* such as in the case of income or return distribution. In the context of the present analysis, because the distribution of outcomes refers to the loss with forecast errors, the “best outcome” case (i.e., dominant case) corresponds to a forecast combination with the largest loss above a given level *z*.

6 Forecasts from different models are updated recursively by expanding the estimation window by one observation forward, thereby reducing the pseudo-out-of-sample test window by one period. Therefore, for each of *h* -step forecasts, we calculate $N_{\!f}$ forecasts from each of the model, as explained in the following section.

7 The asymptotic distribution of $\hat{F}$ is given by $\sqrt{N_{\!f}} (\hat{F}-F)$, which tends weakly to a mean zero Gaussian process $B\circ F$ in the space of continuous functions on $R^{n}$ (see, e.g., the multivariate functional central limit theorem for stationary strongly mixing sequences stated in Rio (2000)).

8 In this paper, we only test first-order SDE in the empirical applications below. Because there are forecast combinations with alternative weighting schemes that dominate the equally weighted forecast combination at the first order, we do not move to the second one.

9 The daily noon buying rates in New York City certified by the Federal Reserve Bank of New York for customs and cable transfer purposes are obtained from the FREDÂ® Economic Data system of Federal Reserve Bank of St. Louis (http://research.stlouisfed.org). The weekly series is generated by selecting the Wednesday series (if Wednesday is a holiday, then the subsequent Thursday is used).

10 Deciding whether the direct or the iterated approach is better is an empirical matter because it involves a trade-off between the estimation efficiency and the robustness-to-model misspecification, see Elliott and Timmermann (2008). Marcellino et al. (2006) have addressed these points empirically using a dataset of 170 US monthly macroeconomic time series. They have found that the iterated approach generates the lowest MSE values, particularly if lengthy lags of the variables are included in the forecasting models and if the forecast horizon is long.

11 This process involves replacing $y_{t}$ with $y_{t+h}$ on the left-hand side of Eq. (9) and running the regression using data up to time *t* to fitted values for corresponding forecasts.

12 Indeed, $d_{t}$ is convex in $y_{t-1}$ whenever $y_{t-1}<c$, and $-d_{t}$ is convex whenever $y_{t-1}>c$. Therefore, by Jensen’s inequality, naive estimation underestimates $d_{t}$ if $y_{t-1}<c$ and overestimates $d_{t}$ if $y_{t-1}>c$.

13 A detailed exposition of approaches for forecasting from a SETAR model can be found in Van Dijk et al. (2003).

14 See Franses and Franses and van Dijk (2000) for a review of feed-forward-type neural network models.

15 In this paper, we only report optimal forecast combinations for 50th, 75th, and 95th percentiles of the error distribution. However, the SDE methodology can also be used to obtain optimal forecast combinations at lower percentiles of the distribution. We do not report these results to conserve space, given that the practical gains of optimal forecast combination at lower percentiles may not be as are important.

16 The empirical distribution of loss consists of different levels of loss, possibly exceeding 150 depending on the nature of the application. Therefore, rather than reporting the optimal forecast combination for all levels of loss, we only report results at selected percentiles of the loss function. However, the full set of optimal forecast combinations for different loss levels can be obtained upon request from authors.

17 In the exchange rate application, over-forecasting or under-forecasting (forecasts that are above and below the realization, respectively) would lead to decisions that would harm the traders. For example, over-prediction (predicting appreciation of foreign currency) could reinforce investors to sell short the domestic currency (and buy foreign currency now, which is forecasted to appreciate in future). Similarly, under-prediction (predicting depreciation of foreign currency) can lead to a short-selling of the foreign currency (i.e., selling the foreign currency now and trading it back in near future). Both over- and under-forecasting would lead to decisions that would harm the traders and hence the trader would aim to minimize the forecast errors rather than the sign of the error and they would not worry about whether the errors have all the same sign. However, given the context of the application, it is possible that the sign of the errors might be important to take into account. We thank the one of the anonymous referees for pointing out this issue.

18 Note that we do not present the individual out-of-sample results of the univariate models, but all of the univariate forecast models perform worse than the equally weighted forecast combination and therefore worse than the forecast combination obtained with the SDE methodology at the 50th, 75th, and 95th percentiles of the error distribution.

19 We also obtained the results for different forecast horizons (${\small h=26}$ and ${\small h=52}$) and the results obtained with the SDE and its relative performance compared to other forecast selection and combination methods remains the same at the 50th, 75th, and 95th percentile of the squared forecast error distribution.

## References

[CR1] Agliardi E, Agliardi R, Pinar M, Stengos T, Topaloglou N (2012). A new country risk index for emerging markets: a stochastic dominance approach. J Empir Finance.

[CR2] Agliardi E, Pinar M, Stengos T (2014). A sovereign risk index for the Eurozone based on stochastic dominance. Finance Res Lett.

[CR3] Agliardi E, Pinar M, Stengos T (2015). An environmental degradation index based on stochastic dominance. Empir Econ.

[CR4] Al-Qassam MS, Lane JA (1989). Forecasting exponential autoregressive models of order 1. J Time Ser Anal.

[CR5] Aiolfi M, Capistrán C, Timmermann A, Clements MP, Hendry DF (2011). Forecast combination. The Oxford handbook of economic forecasting.

[CR6] Anderson G (1996). Nonparametric tests of stochastic dominance in income distributions. Econometrica.

[CR7] Barrett GF, Donald SG (2003). Consistent tests for stochastic dominance. Econometrica.

[CR8] Bates JM, Granger CWJ (1969). The combination of forecasts. Oper Res Q.

[CR9] Beach C, Davidson R (1983). Distribution-free statistical inference with Lorenz curves and income shares. Rev Econ Stud.

[CR10] Burnham KP, Anderson DR (2002). Model selection and multimodel inference: a practical information-theoretic approach.

[CR11] Clemen RT (1989). Combining forecasts: a review and annotated bibliography. Int J Forecast.

[CR12] Clements MP, Galvão AB, Kim JH (2008). Quantile forecasts of daily exchange rate returns from forecasts of realized volatility. J Empir Finance.

[CR13] Clements MP, Hendry DF (1998). Forecasting economic time series.

[CR14] Clements MP, Hendry DF (1999). Forecasting non-stationary economic time series.

[CR15] Clements MP, Hendry DF, Elliott G, Granger CWJ, Timmermann A (2006). Forecasting with breaks. Handbook of economic forecasting.

[CR16] Davidson R, Duclos J-Y (2000). Statistical inference for stochastic dominance and for the measurement of poverty and inequality. Econometrica.

[CR17] Diebold FX, Pauly P (1987). Structural change and the combination of forecasts. J Forecast.

[CR18] De Gooijer JG, De Bruin PT (1998). On forecasting SETAR processes. Stat Probab Lett.

[CR19] Drechsel K, Maurin L (2010). Flow of conjunctural information and forecast of euro area economic activity. J Forecast.

[CR20] Elliott G, Timmermann A (2004). Optimal forecast combinations under general loss functions and forecast error distributions. J Econom.

[CR21] Elliott G, Timmermann A (2005). Optimal forecast combination under regime switching. Int Econ Rev.

[CR22] Elliott G, Timmermann A (2008). Economic forecasting. J Econ Lit.

[CR23] Fernández C, Ley E, Steel MF (2001). Model uncertainty in cross-country growth regressions. J Appl Econom.

[CR24] Fishburn PC (1977). Mean-risk analysis with risk associated with below-target returns. Am Econ Rev.

[CR25] Franses P, van Dijk D (2000). Nonlinear time series models in empirical finance.

[CR26] Gerlach RH, Chen CWS, Chan NYC (2011). Bayesian time-varying quantile forecasting for value-at-risk in financial markets. J Bus Econ Stat.

[CR27] Geweke J, Amisano G (2011). Optimal prediction pools. J Econom.

[CR28] Geweke J, Amisano G (2012). Prediction with misspecified models. Am Econ Rev.

[CR29] Giacomini R, Komunjer I (2005). Evaluation and combination of conditional quantile forecasts. J Bus Econ Stat.

[CR30] Granger CWJ (1999). Outline of forecast theory using generalized cost functions. Span Econ Rev.

[CR31] Granger CWJ, Ramanathan R (1984). Improved methods of combining forecasts. J Forecast.

[CR32] Hamilton J (1994). Time series analysis.

[CR33] Hendry DF, Clements MP (2004). Pooling of forecasts. Econom J.

[CR34] Huang H, Lee T-H (2010). To combine forecasts or to combine information?. Econom Rev.

[CR35] Hyndman RJ, Koehlerb AB (2006). Another look at measures of forecast accuracy. Int J Forecast.

[CR36] Kock AB, Terasvirta T, Clements MP, Hendry DF (2011). Forecasting with nonlinear time series models. Oxford handbook of economic forecasting.

[CR37] Koenker R (2005). Quantile regression.

[CR38] Kolassa S (2011). Combining exponential smoothing forecasts using Akaike weights. Int J Forecast.

[CR39] Lin JL, Granger CWJ (1994). Forecasting from non-linear models in practice. J Forecast.

[CR40] Linton O, Maasoumi E, Whang Y-J (2005). Consistent testing for stochastic dominance under general sampling schemes. Rev Econ Stud.

[CR41] Marcellino M, Stock JH, Watson MW (2006). A comparison of direct and iterated multistep AR methods for forecasting macroeconomic time series. J Econom.

[CR42] Nowotarski J, Raviv E, Trück S, Weron R (2014). An empirical comparison of alternative schemes for combining electricity spot price forecasts. Energy Econ.

[CR43] Patton AJ, Timmermann A (2007). Properties of optimal forecasts under asymmetric loss and nonlinearity. J Econom.

[CR44] Pinar M (2015). Measuring world governance: revisiting the institutions hypothesis. Empir Econ.

[CR45] Pinar M, Stengos T, Topaloglou N (2013). Measuring human development: a stochastic dominance approach. J Econ Growth.

[CR46] Pinar M, Stengos T, Yazgan ME (2015). Measuring human development in the MENA region. Emerg Mark Finance Trade.

[CR47] Post T (2003). Empirical tests for stochastic dominance efficiency. J Finance.

[CR48] Raftery AE (1995). Bayesian model selection in social research. Sociol Methodol.

[CR49] Rio E (2000). Theorie asymptotique des processus aleatoires faiblement dependants.

[CR50] Rissanen J (1986). Order estimation by accumulated prediction errors. J Appl Probab.

[CR51] Sala-i-Martin X, Doppelhofer G, Miller RI (2004). Determinants of long-term growth: a Bayesian averaging of classical estimates (BACE) approach. Am Econ Rev.

[CR52] Scaillet O, Topaloglou N (2010). Testing for stochastic dominance efficiency. J Bus Econ Stat.

[CR53] Smith J, Wallis KF (2009). A simple explanation of the forecast combination puzzle. Oxf B Econ Stat.

[CR54] Stock JH, Watson MW (1999). Forecasting inflation. J Monet Econ.

[CR55] Stock JH, Watson MW, Engle RF, White H (1999). A comparison of linear and nonlinear models for forecasting macroeconomic time series. Cointegration, causality and forecasting.

[CR56] Stock JH, Watson MW (2004). Combination forecasts of output growth in a seven-country data set. J Forecast.

[CR57] Swanson NR, Zeng T (2001). Choosing among competing econometric forecasts: regression-based forecast combination using model selection. J Forecast.

[CR58] Taylor JW, Bunn DW (1998). Combining forecast quantiles using quantile regression: investigating the derived weights, estimator bias and imposing constraints. J Appl Stat.

[CR59] Terasvirta T, Elliott G, Granger CWJ, Timmermann A (2006). Forecasting economic variables with nonlinear models. Handbook of economic forecasting.

[CR60] Timmermann A, Elliott G, Granger CWJ, Timmermann A (2006). Forecast combinations. Handbook of economic forecasting.

[CR61] Van Dijk D, Franses PH, Clements MP, Smith J (2003). On SETAR non-linearity and forecasting. J Forecast.

[CR62] White H, Elliott G, Granger CWJ, Timmermann A (2006). Approximate nonlinear forecasting methods. Handbook of economic forecasting.

